# The Nutritional Supplement *L*-Alpha Glycerylphosphorylcholine Promotes Atherosclerosis

**DOI:** 10.3390/ijms222413477

**Published:** 2021-12-15

**Authors:** Zeneng Wang, Jennie Hazen, Xun Jia, Elin Org, Yongzhong Zhao, Lucas J. Osborn, Nisreen Nimer, Jennifer Buffa, Miranda K. Culley, Daniel Krajcik, Bert-Jan H. van den Born, Koos Zwinderman, Bruce S. Levison, Max Nieuwdorp, Aldons J. Lusis, Joseph A. DiDonato, Stanley L. Hazen

**Affiliations:** 1 Department of Cardiovascular and Metabolic Sciences, Lerner Research Institute, Cleveland Clinic, Cleveland, OH 44195, USA; Hazenje@wustl.edu (J.H.); xxj8@case.edu (X.J.); zhaoy@iscb.org (Y.Z.); osbornl@ccf.org (L.J.O.); nimern278@gmail.com (N.N.); BUFFAJ@ccf.org (J.B.); mkc53@pitt.edu (M.K.C.); KRAJCID@ccf.org (D.K.); DIDONAJ@ccf.org (J.A.D.); HAZENS@ccf.org (S.L.H.); 2 Department of Molecular Medicine, Cleveland Clinic Lerner College of Medicine of Case Western Reserve University, Cleveland, OH 44195, USA; 3 Estonian Genome Centre, Institute of Genomics, University of Tartu, 51010 Tartu, Estonia; elin.org@ut.ee; 4 Department of Vascular Medicine, Academic Medical Center, University of Amsterdam, Meibergdreef 9, 1105 AZ Amsterdam, The Netherlands; b.j.vandenborn@amsterdamumc.nl (B.-J.H.v.d.B.); a.h.zwinderman@amsterdamumc.nl (K.Z.); m.nieuwdorp@amsterdamumc.nl (M.N.); 5 Department of Medicine, Division of Cardiology, University of California, Los Angeles, CA 90095, USA; JLusis@mednet.ucla.edu; 6 Department of Cardiovascular Medicine, Heart, Vascular and Thoracic Institute, Cleveland Clinic, Cleveland, OH 44195, USA

**Keywords:** *L*-alpha glycerylphosphorylcholine, atherosclerosis, trimethylamine, trimethylamine N-oxide, microbiota

## Abstract

*L*-alpha glycerylphosphorylcholine (GPC), a nutritional supplement, has been demonstrated to improve neurological function. However, a new study suggests that GPC supplementation increases incident stroke risk thus its potential adverse effects warrant further investigation. Here we show that GPC promotes atherosclerosis in hyperlipidemic *Apoe*^−/−^ mice. GPC can be metabolized to trimethylamine N-oxide, a pro-atherogenic agent, suggesting a potential molecular mechanism underlying the observed atherosclerosis progression. GPC supplementation shifted the gut microbial community structure, characterized by increased abundance of *Parabacteroides*, *Ruminococcus*, and *Bacteroides* and decreased abundance of *Akkermansia*, *Lactobacillus*, and *Roseburia*, as determined by 16S rRNA gene sequencing. These data are consistent with a reduction in fecal and cecal short chain fatty acids in GPC-fed mice. Additionally, we found that GPC supplementation led to an increased relative abundance of choline trimethylamine lyase (*cutC*)-encoding bacteria via qPCR. Interrogation of host inflammatory signaling showed that GPC supplementation increased expression of the proinflammatory effectors CXCL13 and TIMP-1 and activated NF-κB and MAPK signaling pathways in human coronary artery endothelial cells. Finally, targeted and untargeted metabolomic analysis of murine plasma revealed additional metabolites associated with GPC supplementation and atherosclerosis. In summary, our results show GPC promotes atherosclerosis through multiple mechanisms and that caution should be applied when using GPC as a nutritional supplement.

## 1. Introduction

*L*-alpha glycerylphosphorylcholine (GPC) is a substrate for the synthesis of phosphatidylcholine (PC) and the neurotransmitter acetylcholine in the brain [1,2]. GPC is abundant in various dietary sources, especially in animal products such as meat and fish [3]. Several in vitro studies suggest that PC can be degraded by pancreatic phospholipase A1 to produce GPC [4,5,6,7]. Consequently, as a major component of the mammalian cell membrane [8], PC represents a source of GPC in an omnivorous diet. To date, GPC has been used as a nutritional supplement for the treatment of neurological disorders such as Alzheimer’s disease and dementia, where it has been reported to improve memory, cognitive function, and reverse amnesia in experimental models [9,10]. Additionally, some evidence suggests that GPC improves physical and psychomotor performance in the context of human strength and conditioning [11,12]. However, a new study from a cohort of more than 12 million human subjects indicated that the use of GPC was associated with a higher 10-year incident stroke risk in a dose-response manner [13]. Considering these recent findings, the adverse effect of GPC warrant further investigation.

As a dietary nutrient, the GPC molecule includes a trimethylamine (TMA) moiety. To date, multiple TMA containing nutrients such as choline, carnitine, and γ-butyrobetaine have been confirmed to promote atherosclerosis via the trimethylamine N-oxide (TMAO) metaorganismal pathway, where gut microbiota-derived TMA is oxidized by hepatic flavin monooxygenases (FMOs) to produce TMAO [14,15,16]. TMAO has been shown to modulate multiple proatherogenic effectors, including NF-κB, MAPK signaling, NLRP3 inflammasome activation, and increases pro-inflammatory monocyte abundance in circulating blood [14,15,16,17,18,19]. Given the numerous clinical studies linking TMAO levels to cardiovascular disease risks [14,20,21] and the numerous mechanistic studies linking TMAO to cardiovascular disease pathogenesis [17,22,23], in the present study, we sought to use atherosclerosis prone mice to test the following: (1) whether dietary GPC promotes atherosclerosis, (2) whether GPC can shift gut microbial community structure and the plasma metabolome, and (3) whether GPC alone can activate inflammation associated signaling pathways.

## 2. Results

### 2.1. Gut Microbiota Can Metabolize GPC to Produce TMA

**Figure 1:**
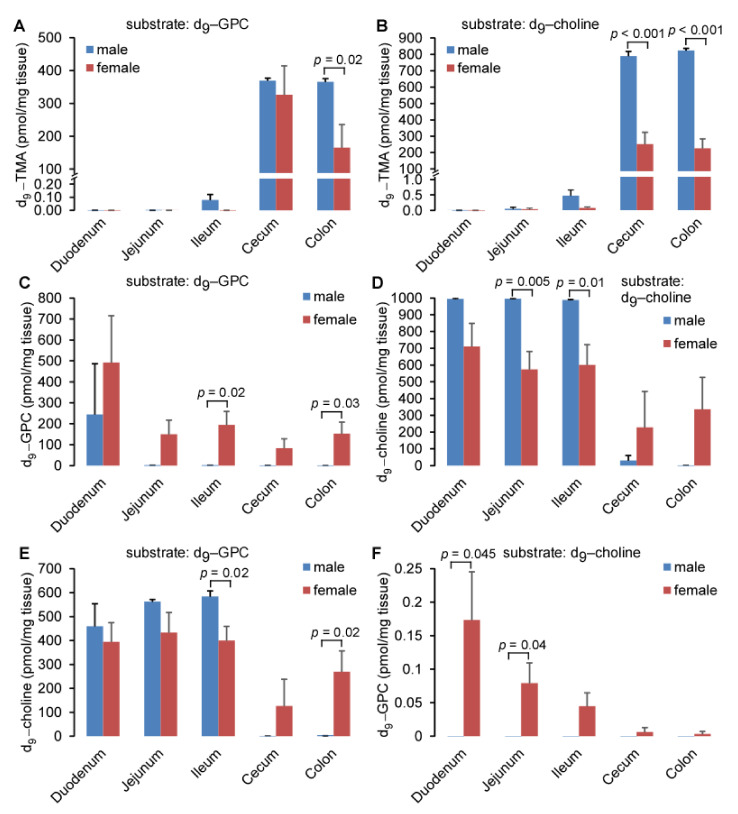
GPC can be efficiently metabolized to TMA and choline after incubation with intestinal segments. (**A**,**B**) d_9_-TMA, (**C**,**F**) d_9_-GPC, and (**D**,**E**) d_9_-choline content after intestinal segments were incubated with 20 volumes of 50 µM d_9_-GPC (**A**,**C**,**E**) or d_9_-choline (**B**,**D**,**F**). Different intestinal segments were harvested from five female or three male C57/BL6J mice and incubated with d_9_-choline and d_9_-GPC under anaerobic conditions for 16 h. d_9_-TMA, d_9_-GPC, and d_9_-choline were quantified by LC-MS/MS, as described in Materials and Methods. Data are presented as mean ± SE from the indicated numbers of mice. *p* values were calculated by Student’s test, and only the values less than 0.05 are provided.

Structurally, *L*-alpha glycerylphosphorylcholine (GPC) contains a trimethylamine (TMA) moiety. In order to test whether polymicrobial communities possessed the capacity to metabolize GPC to TMA, the precursor for trimethylamine N-oxide (TMAO), we incubated different murine intestinal segments with deuterium labeled GPC, GPC-(trimethyl-d_9_) (d_9_-GPC), under anaerobic conditions. Sixteen hours later, the reaction was stopped to quantify deuterium labeled TMA production (d_9_-TMA), where we observed that incubation of the ileum, cecum, and colon leads to the production of d_9_-TMA (Figure 1A). No d_9_-TMA was detected after incubation with the duodenum or jejunum, which is consistent with previous reports that TMA producing bacteria, which tend to be anaerobes, are enriched in the more distal intestine [20]. In parallel, we incubated different intestinal segments with isotope labeled choline, choline-(trimethyl-d_9_) (d_9_-choline). Here we observed that the product, d_9_-TMA, was mainly produced in the cecum and colon (Figure 1B), and that the concentrations were comparable to those of the d_9_-GPC substrate in female mice. In male mice, the addition of d_9_-choline led to double the production of d_9_-TMA compared to d_9_-GPC. Low concentrations of d_9_-TMA were detected in the jejunum and ileum after incubation with d_9_-choline, suggesting bacterial discrimination between GPC and choline when cleaving the C-N bond. Additionally, we quantified d_9_-choline and d_9_-GPC in the intestinal segments after incubation with d_9_-GPC and d_9_-choline, as shown in Figure 1C–F. Interestingly, after incubation with d_9_-GPC or d_9_-choline, residual substrate remained. In the duodenum, nearly 50% (female) and 25% (male) of d_9_-GPC was not metabolized. In the more distal intestinal segments, incubation with d_9_-GPC resulted in complete metabolism of the substrate in male mice, and 80–90% metabolism in female mice. When providing d_9_-choline as a substrate, very little metabolism was observed in the duodenum, jejunum, and ileum for male or female mice. Conversely, the cecum and colon appear to be the predominant site of d_9_-GPC and d_9_-choline bacterial metabolism (Figure 1C,D). After quantifying the inter-conversion between d_9_-GPC and d_9_-choline, we observed that ~40–60% of d_9_-GPC was metabolized to d_9_-choline in the duodenum, jejunum, and ileum, and nearly no d_9_-choline was detected in the cecum or colon after incubation with d_9_-GPC in male mice. A small amount (~25% and ~12%, respectively) of d_9_-GPC remained in the colon and cecum of female mice and can be metabolized to d_9_-choline (Figure 1E). Notably, the incubation of d_9_-choline with each intestinal segment revealed that less than 0.1% of d_9_-choline could be used for the synthesis of d_9_-GPC in any given intestinal segment in female mice, and no d_9_-GPC was detected after intestinal segment incubation with d_9_-choline in male mice (Figure 1F). Choline is more efficiently metabolized to TMA in the cecum and colon of male mice compared to female mice which explains why nearly no choline can be detected in male mouse cecum or colon after incubation with GPC (Figure 1E) and no GPC can be detected in male mouse cecum or colon after incubation with choline (Figure 1F).

### 2.2. Choline TMA Lyase Can Catalyze the Cleavage of GPC to Produce TMA

**Figure 2:**
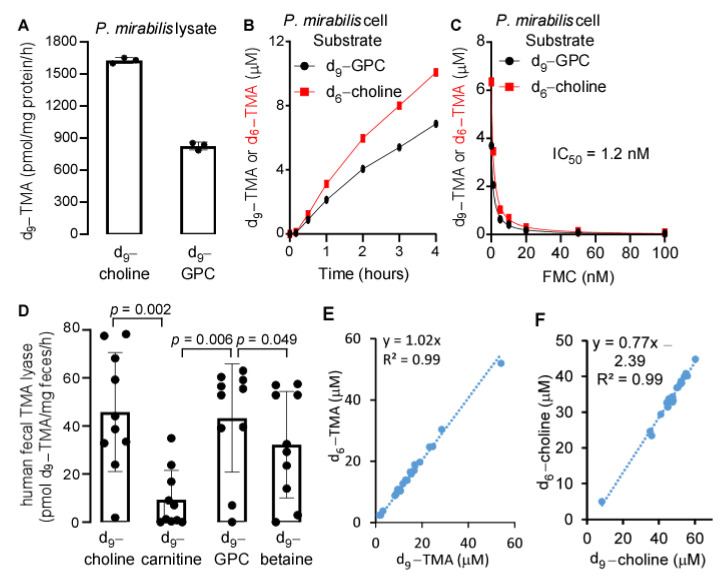
Similarity of GPC to choline as a precursor to produce TMA. (**A**) *Proteus mirabilis* 29906 lysate catalyzed d_9_-TMA production by utilizing d_9_-choline and d_9_-GPC as substrates. Three milligrams of protein equivalents of lysate were used to incubate with 100 µM d_9_-choline or d_9_-GPC in 2 mL PBS at 37 °C for 16 h. (**B**) d_9_-TMA and d_6_-TMA produced from d_9_-GPC and d_6_-choline after incubation with *P. mirabilis* cells at the indicated time, respectively. *P. mirabilis* was grown in LB medium overnight, and the bacterial cells after centrifugation were resuspended in ¼ volume of cold PBS with d_9_-GPC and d_6_-choline added, each at 100 µM. (**C**) Inhibition of d_9_-TMA and d_6_-TMA production from d_9_-GPC and d_6_-choline, respectively, in the presence of different concentrations of fluoromethylcholine (FMC) for 2 h. Bacterial culture and substrate incubation were conducted as described in panel (**B**). (**D**) Human fecal TMA lyase activity utilizing different substrates. Twenty volumes of 100 µM different d_9_-labeled substrates were incubated with fecal tissue for 16 h to quantify d_9_-TMA production by LC-MS. Fecal TMA lyase activity is expressed as pmol d_9_-TMA/mg feces/h. Comparison between substrates was conducted by Wilcoxon matched-pairs signed rank test, where *p* < 0.05 is considered significant. (**E**) Correlation of d_6_-TMA to d_9_-TMA after mouse cecal contents were incubated with 100 µM d_9_-GPC and 100 µM d_6_-choline at the same time for 16 h under anaerobic conditions, *n* = 23. (**F**) Correlation of d_6_-choline to d_9_-choline after cecal contents were incubated with d_6_-choline and d_9_-GPC, as described in panel **E**. d_9_-TMA and d_6_-TMA were quantified by LC-MS/MS, as described in Materials and Methods, and d_6_-choline and d_9_-choline were quantified by LC-MS/MS after being filtered through a 3K-cut off filter with d_4_-choline added as internal standard. Data are presented as mean ± SD from three replicates in panel (**A**), two replicates in panels (**B**,**C**), and ten replicates in panel (**D**), with scatter plots in panels (**A**,**D**).

Previously, we have shown that the purified bacterial choline TMA lyase, cutC/D, can utilize GPC as a substrate to produce TMA directly [21]. As expected, the bacterial lysate of *Proteus mirabilis*, which encodes CutC/D [22], can also utilize GPC as a substrate to produce TMA (Figure 2A). Additionally, we incubated *P. mirabilis* 29906 with d_9_-GPC and (N,N-dimethyl-d_6_) choline (d_6_-choline) each at a concentration of 100 µM, and monitored d_9_-TMA and d_6_-TMA production over time. Shown in Figure 2B, we observed that both d_9_-TMA and d_6_-TMA production were increased over time. Interestingly, a structural analogue of choline, N-fluoromethylcholine (FMC), which we previously reported as a potent inhibitor of CutC/D [23], inhibited TMA production from both GPC and choline precursors after incubation with *P. mirabilis* (Figure 2C). In order to characterize substrate specificity for human fecal TMA lyase enzymes, we utilized the deuterium labeled nutrients d_9_-choline, d_9_-GPC, d_9_-carnitine, and d_9_-betaine as substrates to monitor the production of d_9_-TMA. Shown in Figure 2D, we observed that the enzymatic utilization of GPC and choline to produce TMA were somewhat similar to each other (Pearson correlation, r^2^ = 0.29, *p* < 0.001). Considering the different bacterial compositions expected in human fecal material and the mouse cecum, we incubated mouse cecal contents simultaneously with d_6_-choline and d_9_-GPC, each at a concentration of 100 µM under anaerobic conditions at 37 °C for 16 h and monitored d_9_-TMA and d_6_-TMA production, respectively. Remarkably, a near perfect linear correlation (slope = 1.02, r^2^ = 0.99) existed between the two products, d_9_-TMA and d_6_-TMA (Figure 2E). Similarly, the remaining substrates, d_6_-choline and d_9_-choline from d_9_-GPC also showed a robust positive correlation (slope = 0.77, r^2^ = 0.99) (Figure 2F), and no GPC was detected after 16 h, which suggests that in the mouse intestine, GPC may be hydrolyzed to choline first followed by subsequent metabolism to yield TMA.

### 2.3. Bacterium Efficiently Metabolizes GPC to Choline

**Figure 3:**
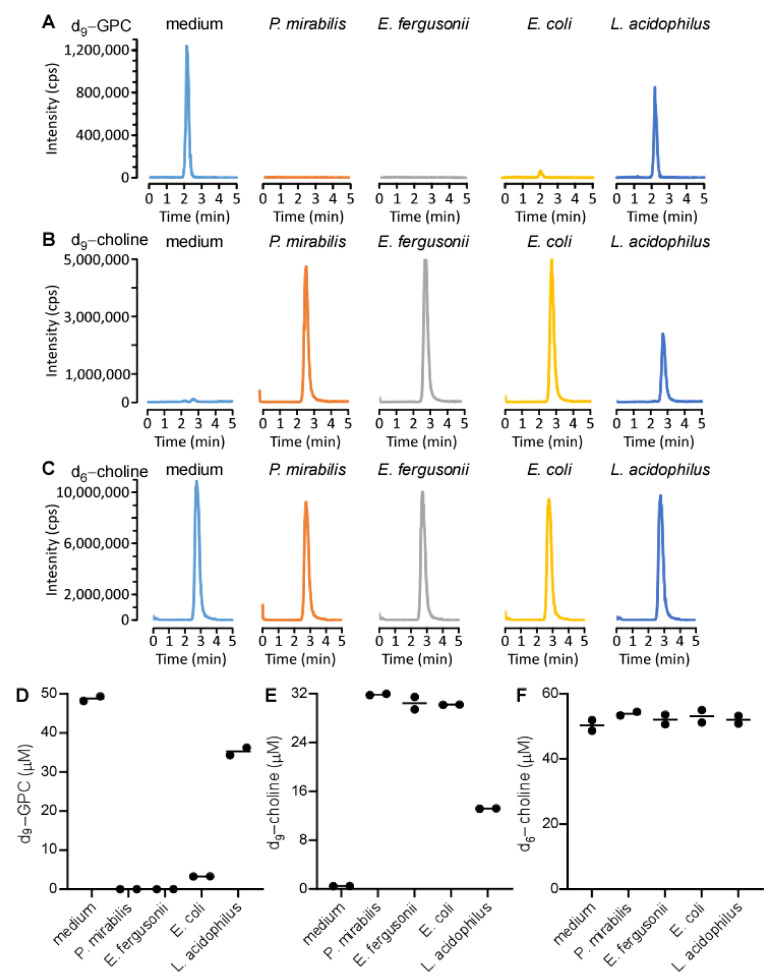
Monitoring product formation after bacterial incubation with deuterated substrates. *P. mirabilis*, *E. fergusonii*, *E. coli* Top10, and *L. acidophilus* were cultured in LB medium overnight. 200 µL of overnight growth suspension was mixed with 200 µL 100 µM d_9_-GPC and d_6_-choline. After 10 min of incubation at 37 °C, bacterial suspensions were filtered through a 3K cutoff membrane, and the filtrate was mixed with d_4_-choline as internal standard to determine the concentrations of d_9_-GPC, d_9_-choline, and d_6_-choline. (**A**–**C**) Ion extracted LC chromatograms in positive-ion multiple reaction monitoring mode with parent to daughter transitions of 267 → 125, 113 → 69, and 110 → 66, corresponding to d_9_-GPC (**A**), d_9_-choline (**B**), and d6-choline (**C**), respectively. (**D**–**F**) concentrations of d_9_-GPC, d_9_-choline, and d_6_-choline, respectively, calculated after bacterial incubation. Each dot in panels (**D**–**F**) denote an independent experiment**.**

To further characterize the bacterial catabolism of GPC, we simultaneously incubated d_9_-GPC and d_6_-choline with various bacteria, including the *cut*C/D encoding bacteria, *P. mirabilis* 29906 and *Escherichia fergusonii*, as well as the *cut*C/D non-encoding bacteria, *Escherichia coli* Top10 (a competent bacterium) and *Lactobacillus acidophilus*. This way, by quantifying both d_9_-TMA and d_6_-TMA, we could distinguish which nutrient precursor gave rise to the TMA isotopologue produced. After incubation, the reaction was stopped and we quantified the precursors and products using targeted liquid chromatography with tandem mass spectrometry (LC-MS/MS). As expected, d_9_-GPC disappeared after incubation with *P. mirabilis* 29906 and *E. fergusonii* (Figure 3A). Interestingly, a small amount of d_9_-GPC was detected after incubation with *E. coli* Top10, and over 50% of the initial d9-GPC substrate was detected after incubation with *L. acidophilus*. The concentration of d_9_-choline product was in concordance with the amount of d_9_-GPC consumed for each bacterium (Figure 3B). There was no observable difference in d_6_-choline levels across any of the conditions (Figure 3C). These results were further confirmed by adding a stable isotope labeled choline, choline-1,1,2,2-d_4_ (d_4_-choline), as an internal standard to quantify the concentrations of d_9_-GPC, d_9_-choline, and d_6_-choline by LC-MS/MS (Figure 3D–F).

### 2.4. GPC Is Abundant in the Mouse Gastrointestinal Tract

**Figure 4:**
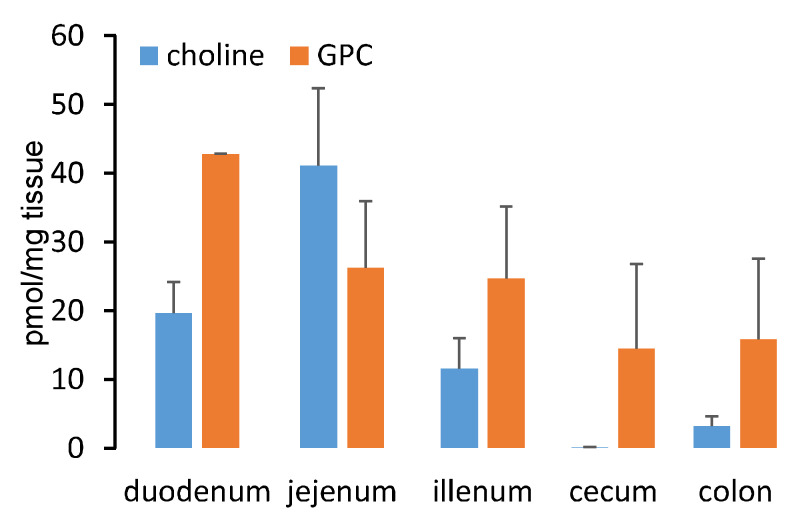
GPC is abundant in the gut. GPC and choline distribution in the mouse gut. Four female C57BL/6J mice were sacrificed and the gut was collected to quantify GPC and choline by LC-MS. Data are presented as mean ± SE.

We next measured the distribution of GPC throughout the mouse intestinal lumen (Figure 4), where we observed that GPC was most abundant in the duodenum (consistent with where endogenous pancreatic lipases are introduced into the intestines) and there existed a decreasing gradient of luminal GPC content from proximal to distal segments. Choline was also detected in the intestine at concentrations comparable to GPC in the duodenum, jejunum, and ileum. Unlike GPC, choline was present only in trace amounts in the mouse cecum.

### 2.5. Oral Gavage of GPC Increases Circulating TMAO Levels

**Figure 5:**
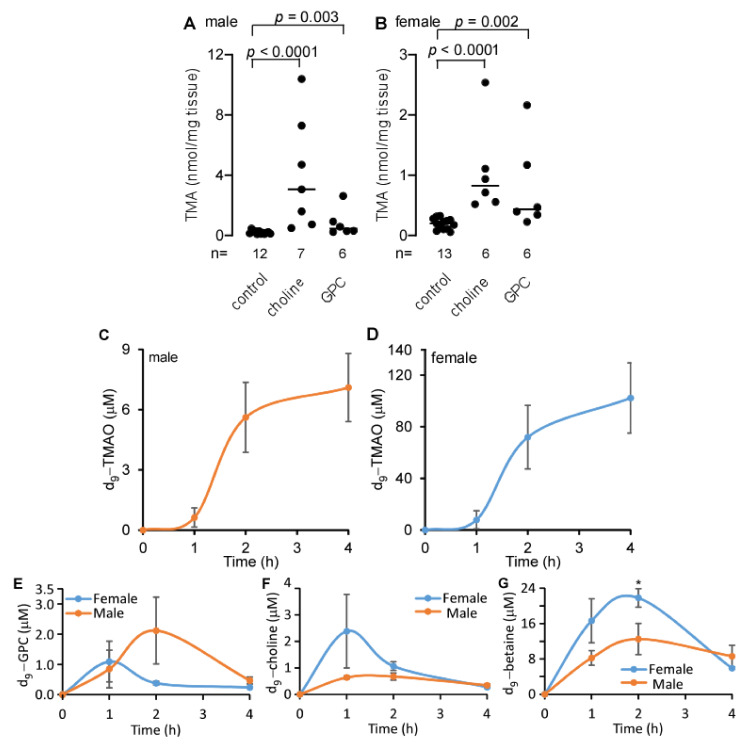
GPC supplementation increases cecal TMA and plasma TMAO production. (**A**,**B**) Cecal TMA content in *LDLr*^−/−^ male (**A**) and female (**B**) mice fed choline, GPC supplemented chow diet, and control chow diet for one month. *p* values were calculated by Wilcoxon rank sum test. (**C**,**D**) Production of d_9_-TMAO in plasma of *Apoe*^−/−^ male (**C**) and female (**D**) mice after oral gavage with 150 µL 150 mM d_9_-GPC. (**E**–**G**) Plasma d_9_-GPC, d_9_-choline, and d_9_-betaine in *Apoe*^−/−^ male and female mice after oral gavage with 150 µL 150 mM d_9_-GPC. Data are presented as mean ± SE from five male and five female mice in each group (**C**–**G**). * *p* < 0.05 for comparison between male and female mice, determined by Student’s *t* test.

*LDLr*^−/−^ mice, a human-relevant animal model of atherosclerosis with deficient endocytosis of circulating LDL [24], fed a GPC or choline supplemented chow diet, had a significantly higher concentration of TMA in the cecum when compared to control chow-fed mice (Figure 5A,B). To test whether GPC can contribute to circulating TMAO levels, we challenged apolipoprotein E knockout (C57BL/6J *Apoe*^−/−^) mice. These mice are commonly used as an animal model of atherosclerosis due to their poor lipoprotein clearance and subsequent accumulation of cholesterol ester-enriched particles in the blood, which leads to the development of atherosclerotic lesions similar to those found in humans [25,26]. We provided *Apoe*^−/−^ mice with a single oral gavage of isotope labeled GPC, d_9_-GPC (150 µL, 150 mM per mouse), and collected blood after 1, 2, and 4 h. Shown in Figure 5C,D, d_9_-TMAO production increased postprandially over time. These data suggest that GPC contributes to the levels of TMAO in peripheral blood. Notably, female mice had much higher levels of d_9_-TMAO following d_9_-GPC gavage when compared to male mice. These data are consistent with our previously reported finding that female mice have higher flavin monooxygenase (FMO) 3 expression, the most abundant and TMA-specific FMO member, than male mice [14,27]. In addition to d_9_-TMAO production, we also observed d_9_-GPC, d_9_-choline, and d_9_-betaine in circulating blood gavaged with d_9_-GPC (Figure 5E–G). Comparing differences in the plasma concentrations of d_9_-GPC, d_9_-choline, and d_9_-betaine at the indicated time points between male and female mice, we observed that only two hours after d_9_-GPC gavage, female mice have significantly higher concentrations of d_9_-betaine than male mice. Notably, this observed difference is less than two-fold, which is far lower than the fold change in plasma d_9_-TMAO between male and female mice. We did not note other statistically significant differences in Figure 5E–G.

### 2.6. Dietary GPC Promotes Atherosclerosis in Apoe^−/−^ Mice

**Figure 6:**
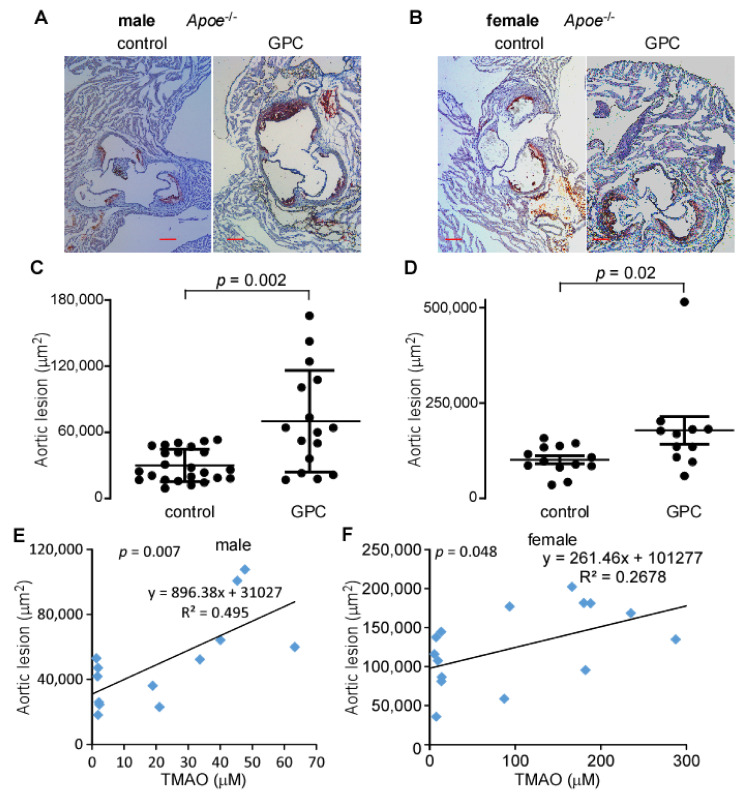
GPC promotes atherosclerosis. (**A**,**B**) Representative Oil-red-O/haematoxylin-stained aortic root sections from male (**A**) and female (**B**) C57BL/6J *Apoe*^−/−^ mice fed control or GPC supplemented chow diets, scale bar = 0.2 mm. (**C**,**D**) Aortic lesion area in 20-week-old male (**C**) and female (**D**) C57BL/6J *Apoe*^−/−^ mice fed GPC supplemented chow diet versus control chow diet. *p* values were calculated by Wilcoxon rank sum test. (**E**,**F**) Pearson correlation of aortic lesion to plasma TMAO in 20-week-old male and female C57BL/6J *Apoe*^−/−^ mice fed either GPC supplemented chow diet or control chow diet for 16 weeks.

The results outlined thus far have confirmed that GPC can be cleaved by microbial enzymes to produce TMA, mostly via a choline intermediate, which is further oxidized in the liver to TMAO. The pro-inflammatory and pro-atherogenic properties of TMAO have been well documented [15,16,18,28,29,30,31,32,33,34]. To determine whether GPC supplementation similarly enhances atherosclerosis, we fed C57BL/6J *Apoe*^−/−^ mice a chow diet supplemented with 1% GPC or a control chow diet for 16 weeks. Figure 6A,B shows representative images from both male and female mice fed GPC supplemented and control chow diets. The quantified aortic root lesion area from all animals is shown in Figure 6C,D. GPC supplementation resulted in a marked increase in atherosclerotic lesion area, as expected given the marked elevation in circulating TMAO (Table 1). Moreover, plasma TMAO positively correlated with aortic lesion area in both sexes (Figure 6E,F). Of note, GPC did not alter the plasma lipid profile, blood sugar, or circulatory markers of renal and hepatic function (Table 1). Taken together, these data support the hypothesis that GPC supplementation promotes atherosclerosis, presumably by acting as a nutrient precursor to TMAO. These findings are consistent with previous reports that other dietary molecules such as choline, carnitine, and γ-butyrobetaine serve as precursory substrates for TMAO production and subsequent atherosclerosis [14,15,16].

### 2.7. GPC Decreases Cecal and Fecal Short Chain Fatty Acids in LDLr^−/−^ Mice

Short chain fatty acids (SCFAs), such as acetic acid, propionic acid, and butyric acid, are known gut microbial metabolites resulting from the fermentation of dietary fiber [35,36]. SCFAs have been reported to act as anti-inflammatory agents, modulators of immunity, and alleviators of some diseases [37]. In vascular endothelial cells, the SCFAs receptors GPR41 and GPR43 have been shown to bind butyrate [38]. This interaction in turn confers a protective effect against endothelial dysfunction, an early step in the progression of atherosclerosis [38,39].

**Figure 7:**
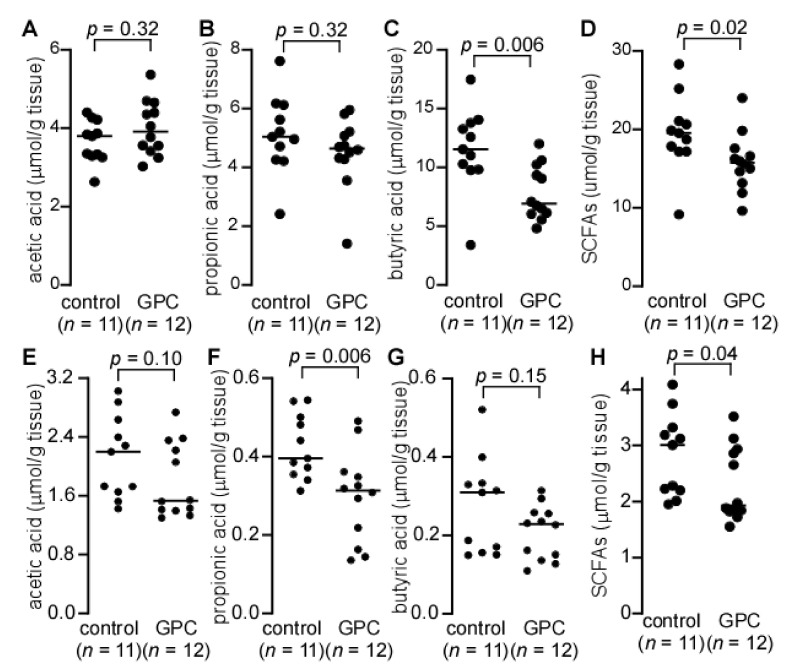
Cecal and fecal short chain fatty acids (SCFAs) in *LDLr*^−/−^ mice fed GPC supplemented or control chow diet. (**A**–**D**) *LDLr*^−/−^ mice were fed GPC supplemented chow diet or control chow diet for one month and cecum was collected to quantify SCFAs. (**E**–**H**) *LDLr*^−/−^ mice were fed GPC supplemented chow diet or control chow diet for three weeks, and feces was collected to quantify SCFAs. Cecum and feces were homogenized in water with isotope labeled SCFAs, [^13^C_2_] acetic acid, [^13^C_3_] propionic acid, and [^13^C_4_] butyric acid added as internal standards. The filtrate was used to quantify SCFAs after derivatization by LC-MS. *p* values were calculated by Wilcoxon rank sum test. Panels (**D**,**H**) display total SCFAs, which is the sum of acetic acid, propionic acid, and butyric acid, from panels (**A**–**C**) and (**E**–**G**), respectively.

To test whether atherosclerosis resulting from GPC supplementation might be related to SCFAs, we measured cecal and fecal levels of SCFAs in *LDLr*^−/−^ mice fed either a control chow diet or GPC-supplemented diet (Figure 7A–H). Here we observed that *LDLr*^−/−^ mice fed GPC supplemented chow had significantly decreased levels of butyric acid in the cecum (Figure 7C) and propionic acid in the feces (Figure 7F). Furthermore, the total SCFAs (acetic acid, propionic acid, and butyric acid) levels were significantly decreased in both the cecum and feces of mice fed GPC supplemented chow when compared to control chow-fed mice (Figure 7D,H).

### 2.8. GPC Shifts the Gut Microbial Community Structure in Apoe^−/−^ Mice

**Figure 8:**
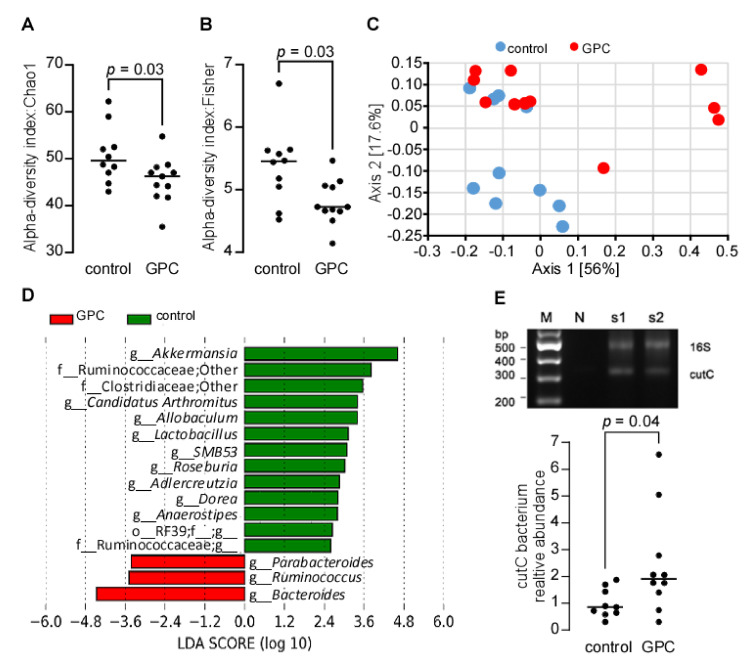
GPC supplementation shifts the gut microbial community. (**A**,**B**) alpha-diversity: Chao1 (**A**) and Fisher index (**B**) of gut microbiota community structure in female C57BL/6J *Apoe*^−/−^ mice after 16 weeks on GPC supplemented (*n* = 11) or control chow diet (*n* = 10). Cecal DNA was extracted for 16S rRNA gene sequencing. (**C**) Principal coordinates analysis of weighted UniFrac distances of microbial community with proportion of variance explained by each principal coordinate axis denoted in each axis label. (**D**) LEfSe identified cecal microbial taxa enriched in GPC-fed mice vs. control chow diet-fed mice (*p* < 0.05 by the Kruskal–Wallis test, log_10_(LDA score) > 2). (**E**) RT-PCR quantitation of choline TMA lyase C subunit (CutC) encoding bacterium relative abundance in female C57BL/6J *Apoe*^−/−^ mice fed either GPC supplemented chow or control chow diet. The relative abundance of CutC-containing bacteria was normalized to that of control chow diet feeding mice. The top panel in (**E**) is the confirmation of specificity for CutC and 16S rRNA gene PCR products from two typical samples by 2% agarose gel electrophoresis with no DNA template as negative control (N) and 100 bp DNA ladder as marker (M). *p* values were calculated by Wilcoxon rank sum test.

GPC decreased cecal butyric acid and fecal propionic acid in *LDL*r^−/−^ mice, suggesting that GPC has the potential to shift the gut microbial community by decreasing the abundance of microbes that can ferment dietary fiber. To test the hypothesis, we performed 16S rRNA gene sequencing of the cecal microbes from female C57BL/6J *Apoe*^−/−^ mice fed a GPC supplemented or control chow diet. We identified a total of 459,625 sequences with an average of 21,887 (*n* = 21) sequences per sample. GPC supplemented chow diet-fed mice had lower alpha diversity in their cecum based on the Chao1 and Fisher indices (Figure 8A,B). We next compared microbial community structure (beta diversity) between GPC supplemented chow and control chow diet-fed mice using Principal Coordinates Analysis (PCoA) transformation of weighted UniFrac distances (Figure 8C). The two groups of mice showed nearly separate Clusters in PCoA space (PERMANOVA, *p* = 0.03). The Linear discriminant analysis Effect Size (LEfSe) revealed increased abundance of the genera *Parabacteroides*, *Ruminococus*, and *Bacteroides* and decreased abundance of *Akkermansia*, *Adlercreutzia*, *Allobaculum*, *Anaerostipes*, *Candidatus Arthromitus*, *Dorea*, *Lactobacillus*, Roseburia, Ruminococcaceae, SMB5, and unknown genera from order RF39 (o-RF39; f__g__) in GPC-fed mice compared to control chow diet-fed mice (Figure 8D). Notably, *Roseburia* sp. are human gut commensals and butyric acid producers—A reduction of which may contribute to various chronic disorders [40,41,42]. In our study, the decreased abundance of *Roseburia* in GPC-fed mice is consistent with the reduced cecal butyric acid, as mentioned above (Figure 7C).

The gut microbial contribution to TMAO production from choline substrates has been clearly established [14]. Here we demonstrate the metaorganismal metabolism of GPC to TMAO—possibly through the same bacterial TMA lyase used for choline metabolism (*cut*C/D). Because some bacterial species harboring *cut*C/D are minimally abundant, 16S rRNA gene sequencing may lack the sensitivity necessary to accurately detect all CutC/D expressing bacteria. To this point, we used a more sensitive method to quantify *cut*C abundance by qPCR using degenerate primers [43], where it was determined that GPC significantly increased the relative abundance of the *cut*C encoding bacteria (Figure 8E).

**Figure 9:**
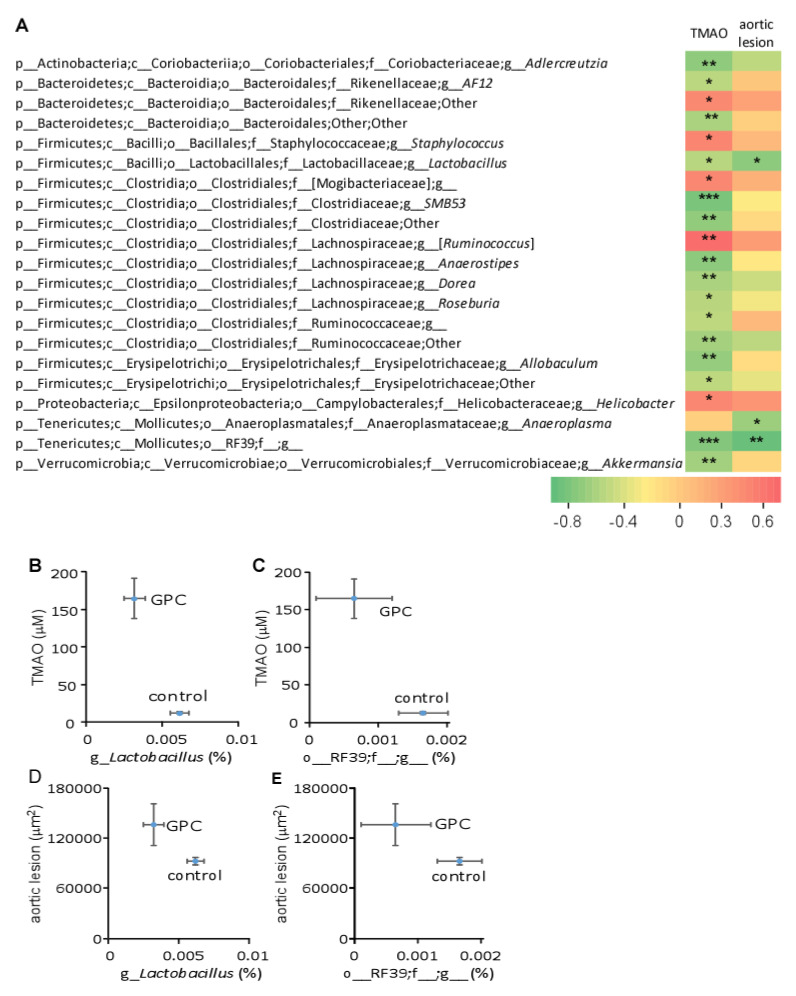
Bacterial taxa in mouse cecum that significantly correlate to either plasma TMAO level or aortic lesion area. (**A**) Spearman correlation heat map demonstrating the association between the indicated microbial taxonomic genera and TMAO concentrations or aortic lesion area. * *p* < 0.05; ** *p* < 0.01; *** *p* < 0.001. (**B**–**E**) GPC supplementation shifts the relative abundance of two genera that significantly correlate with plasma TMAO level or aortic lesion area. Ten female C57BL/6J *Apoe*^−/−^ mice fed control chow diet and 11 female C57BL/6J *Apoe*^−/−^ mice fed GPC supplemented chow diet were utilized.

Further Spearman correlation analysis revealed numerous bacterial taxa significantly associated with plasma TMAO concentration or aortic lesion area (Figure 9A). Because TMAO was strongly correlated with aortic lesion area (Figure 6E,F), some of the bacterial taxa associated with plasma TMAO levels were also highly correlated to aortic lesion area. Figure 9B,C, reveals that GPC supplementation decreased the relative abundance of *Lactobacillus* and o-RF39; f__g__. Strikingly, these same two taxa were negatively correlated with both plasma TMAO concentration and aortic lesion area (Figure 9B–E).

### 2.9. GPC Induces Pro-Inflammatory Cytokine Changes in the Plasma of LDLr^−/−^ Mice

**Figure 10:**
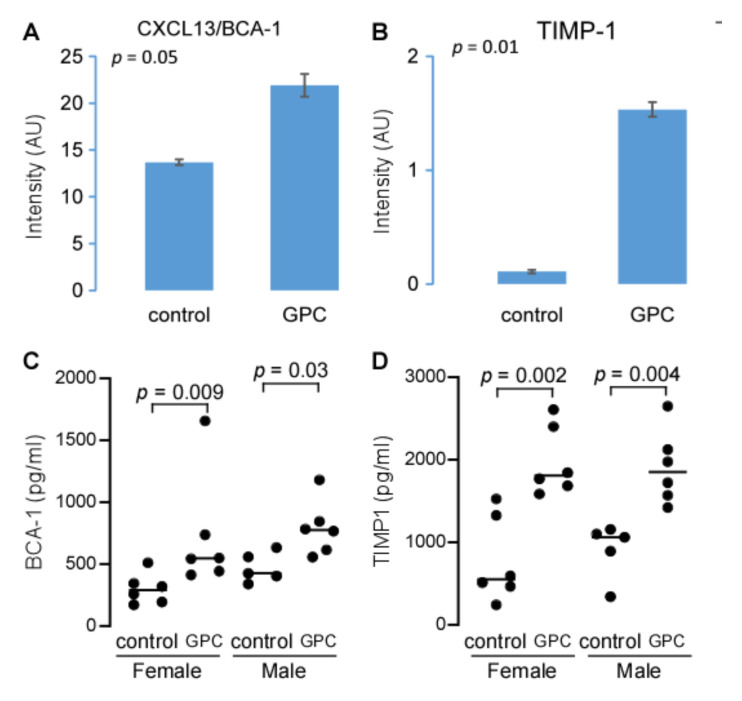
GPC increases expression of the pro-inflammatory chemokine CXCL13/BCA-1 and cytokine TIMP-1. (**A**,**B**) Comparison of plasma CXCL13/BCA-1 (**A**) and TIMP-1 (**B**) expression levels. Plasma was pooled from 10 female C57BL/6J *LDLr*^−/−^ mice fed GPC supplemented chow diet, or 10 female mice fed control chow diet with 10 µL plasma each mouse. CXCL13/BCA-1 (**A**) and TIMP-1 (**B**) were determined by Proteome Profiler^TM^ Array using Mouse Cytokine Array Panel A kit. (**C**,**D**) BCA-1 and TIMP-1 concentrations in plasma were determined by ELISA. Each group contains 5 or 6 C57BL/6J *LDLr*^−/−^ mice fed GPC supplemented chow diet or control chow diet, as indicated, for one month. *p* values in (**A**,**B**) were calculated by Student’s *t* test and in (**C**,**D**) by Wilcoxon rank sum test.

The data above suggest that GPC supplementation promotes atherosclerosis, possibly through the microbe-host co-metabolite TMAO. To further characterize the pro-atherogenic properties of dietary GPC, we used the Proteome Profiler™ Mouse Cytokine Array on plasma samples from *LDLr*^−/−^ mice fed either GPC supplemented or control chow diets. Although 40 different capture antibodies were applied to each film, we only detected quantitative signals from 6 cytokines: sICAM-1, C5/C5a, TIMP-1, SDF-1, CXCL13/BCA-1, and M-CSF. Notably, plasma CXCL13/BCA-1 and TIMP-1 expression levels were increased in GPC-fed mice compared to the control chow-fed mice (Figure 10A,B). To further validate these preliminary findings from pooled plasma, we utilized ELISA assays targeting CXCL13/BCA-1 and TIMP-1. This confirmatory approach corroborated the finding that GPC supplementation increased the plasma concentrations of both CXCL13/BCA-1 and TIMP-1 (Figure 10C,D).

### 2.10. GPC Activates MAPK and NF κB Signaling

**Figure 11:**
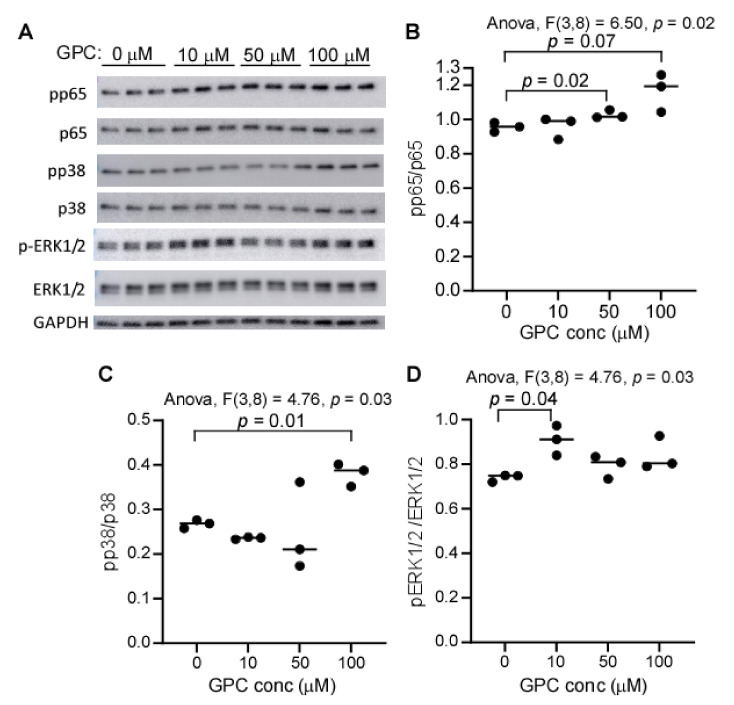
GPC activates NFκB and MAPK signaling in HCAEC. (**A**) SDS-PAGE/Western blotting analysis of ERK1/2, p38, and p65, and their respective phospho-proteins. HCAEC cells were treated with different concentrations of GPC for 20 min, and the lysate was employed to run SDS-PAGE followed by immunoblotting. Each well was loaded with 4 µg protein equivalent of cell lysate. (**B**–**D**) The relative intensity of phospho-protein to its native protein, as quantified by ImageJ software. *p* values were calculated by one-way ANOVA followed by Student’s *t* test, and only *p* values less than 0.10 are provided.

We have previously shown that TMAO can activate MAPK and NFκB signaling in cultured human primary aortic endothelial cells [44]. Given that GPC can be metabolized to TMAO in vivo, we hypothesized that GPC supplementation would activate MAPK and NFκB signaling in the aorta via the microbe-host co-metabolite TMAO. Additionally, we observed that GPC was detected in peripheral blood, and therefore GPC may interact with artery endothelial cells (Figure 5E). To determine if GPC can directly activate MAPK and NFκB signaling, we treated human coronary artery endothelial cells (HCAECs) with various concentrations of GPC for 20 min, at which point cells were harvested and protein lysates were prepared for Western blot analysis. As shown in Figure 11A, GPC treatment led to the activation of p38 MAPK, ERK MAPK, and NFκB signaling, which were further confirmed by band intensity quantification (Figure 11B–D, one-way ANOVA, all *p* < 0.05). Interestingly, GPC treatment led to the dose-dependent phosphorylation of p65, suggesting a potential direct effect of GPC on p65.

### 2.11. GPC Induces a Shift in the Plasma Metabolome of Apoe^−/−^ Mice

**Figure 12:**
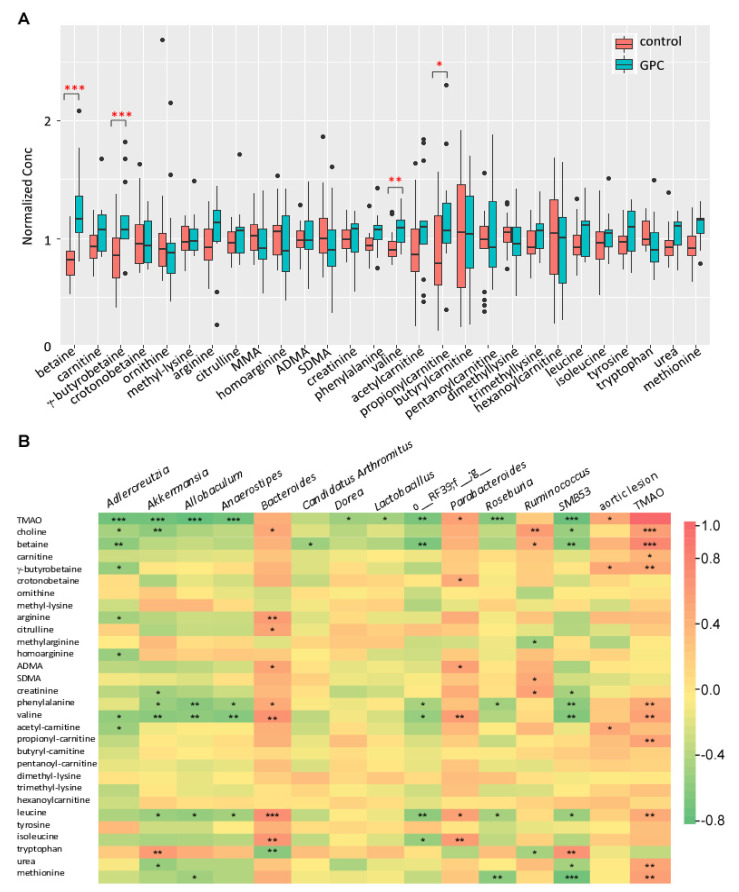
GPC supplementation alters the plasma concentration of gut microbial metabolites implicated in the TMAO pathway. (**A**) Targeted metabolomic profile in plasma in female C57BL/6J *Apoe*^−/−^ mice fed a GPC supplemented chow diet for 16 weeks versus control chow diet. The concentration for each metabolite was normalized to the average concentration in the combined chow and GPC groups. (**B**) Heat map showing Spearman correlation coefficients for the targeted metabolites and the bacterial taxa showing discrimination between the two diet arms as shown in Figure 8D and aortic lesion area and plasma TMAO level. * *p* < 0.05; ** *p* < 0.01; *** *p* < 0.001.

Besides the significant change in plasma TMAO (Table 1), we also noticed that closely related metabolites such as betaine, γ-butyrobetaine, valine, and propionyl-carnitine were significantly altered in *Apoe*^−/−^ mice fed GPC supplemented chow compared to control chow-fed animals (Figure 12A). Additionally, we performed Spearman correlation analysis among plasma metabolites such as TMAO, aortic lesion area, and bacterial taxa which showed discrimination between the two diet arms (Figure 12B). Here we can see that the unknown genera from order RF39 (o_RF39;f_;g_), which is enriched in mice fed control chow diet, negatively correlates with plasma TMAO, betaine, phenylalanine, leucine, isoleucine, and valine concentrations. Remarkably, each of these metabolites, with the exception of isoleucine, is positively correlated to plasma TMAO concentration. Most of the bacterial taxa identified to discriminate GPC supplemented chow versus control chow-fed mice (Figure 8D) were correlated to at least one plasma metabolite. For example, the genus *Akkermansia*, which is enriched in the cecum of mice fed a control chow diet, negatively correlates with plasma TMAO, choline, creatinine, phenylalanine, valine, leucine, and urea, while positively correlating with tryptophan.

**Figure 13:**
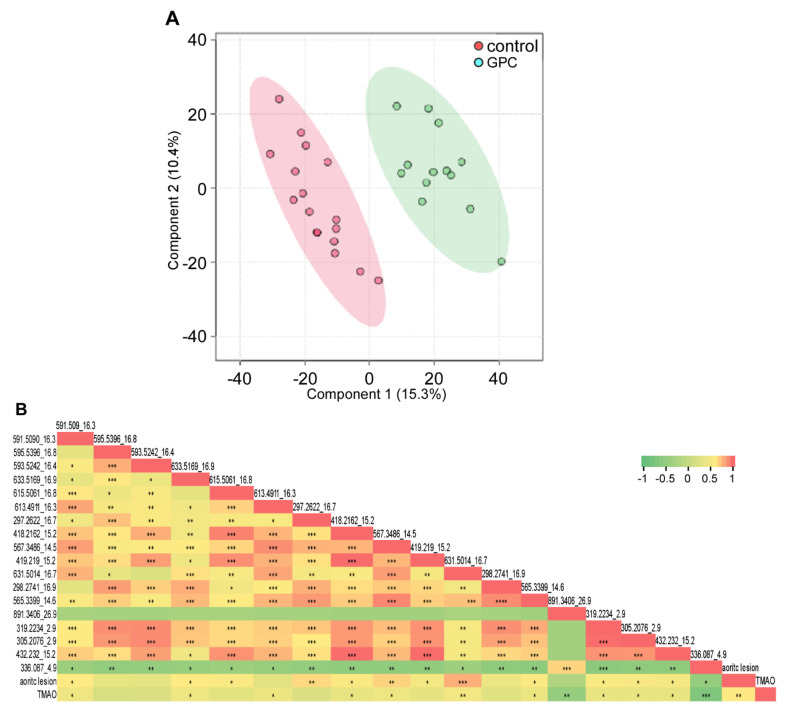
Untargeted metabolomic analysis of plasma metabolites in GPC supplemented chow diet-fed mice versus control chow diet-fed mice. (**A**) PLS-DA of the untargeted metabolomic data in plasma resulted in a clear separation of the metabolic features between female C57BL/6J *Apoe*^−/−^ mice fed GPC supplemented or control chow diets. (**B**) Pearson correlations (r) among the untargeted metabolomic features with fold change larger and FDR smaller than TMAO by comparison of the two groups and plasma TMAO concentration, aortic lesion area. Untargeted metabolomic features in the plasma of female C57BL/6J *Apo*e^−/−^ mice fed GPC supplemented or control chow diet for 16 weeks were acquired by LC-Triple TOF 5600 mass spectrometer using positive IDA mode. The acquired data were analyzed by XCMS to extract ion specific feature labeled with m/z and retention time (rt). Plasma TMAO concentration was determined by stable isotope dilution LC-MS. * *p* < 0.05; ** *p* < 0.01; *** *p* < 0.001.

To further investigate the plasma metabolome in an unbiased manner, we employed an untargeted metabolomic analysis of plasma samples collected from *Apoe*^−/−^ mice fed either a GPC supplemented or control chow diet using an LC-Triple TOF™ 5600 System. In total, we acquired 2917 features; among them, 364 were differentially abundant in mice fed a GPC diet versus a chow control diet (FDR < 0.01). We observed 393 features with a difference of greater than two-fold in mean value between the two diets. The discrimination between diet-induced metabolomic profiles was further characterized by PCA and PLS-DA (2 components, R^2^ = 0.93, Q^2^ = 0.70) (Figure 13A). We collected all other features (18 in total, each feature was labeled as m/z_rt (retention time)) with fold change larger and FDR smaller than TMAO by comparison of the two groups and conducted regression analysis with plasma TMAO and aortic lesion. These results are shown in Figure 13B, where we noted multiple features significantly positively correlated to not only each other, but also to TMAO and aortic lesion. Notable exceptions were 891.3406_26.9, which was significantly negatively correlated to TMAO with no significant correlation to aortic lesion, and 336.087_4.9, which was significantly negatively correlated to both TMAO and aortic lesion. There were three metabolites, 297.2622_16.65, 419.219_15.2, and 615.5062_16.26, that showed significant positive correlation to aortic lesion, but lacked correlation to plasma TMAO.

## 3. Discussion

**Figure 14:**
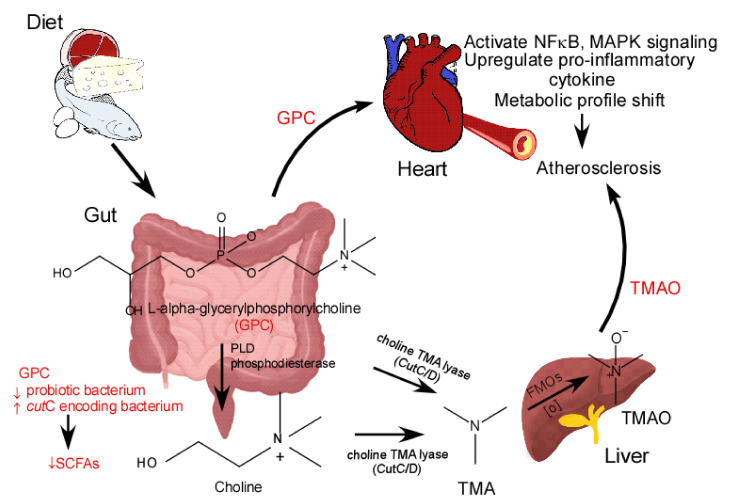
Schematic Figure showing GPC promoting atherosclerosis through multiple mechanisms. PLD, phospholipase D; FMOs, Flavin monooxygenases; SCFAs, short chain fatty acids.

In this study, we show that GPC, a dietary nutrient containing a TMA moiety, promotes atherosclerosis. GPC-induced atherosclerosis appears to be mediated by multiple mechanisms. First, GPC serves as a substrate for the biosynthesis of the pro-atherogenic microbe-host co-metabolite, TMAO. Second, GPC supplementation can shift the gut microbial community by decreasing the relative abundance of SCFA-producing bacteria, leading to reduced SCFA production and elevated levels of pro-inflammatory cytokines and chemokines. Third, GPC can activate MAPK and NFκB signaling in artery endothelial cells, further contributing to the inflammatory dependencies of atherosclerosis. Finally, GPC supplementation leads to alterations in the plasma metabolomic profile, which may be linked to atherosclerosis beyond the TMAO metaorganismal pathway (Figure 14).

The association between TMAO and atherosclerosis has been widely investigated and corroborated on a global scale [45,46,47,48,49,50,51,52,53,54,55,56]. As a pro-atherogenic molecule, TMAO can activate vascular endothelial cell MAPK and NFκB signaling and increase the adhesion of activated monocytes to vascular endothelial cells [18]. Moreover, TMAO has been shown to activate the NLRP3 inflammasome and subsequently release the proinflammatory cytokines IL1-β and IL-18 [31,34]. In this study, we have also shown that GPC can directly activate vascular endothelial cell MAPK and NFκB signaling, which further supports our hypothesis that GPC is implicated in inflammation.

GPC is readily degraded in the gut to produce choline, a substrate for TMAO production. Alternatively, GPC can be cleaved at the C-N bond to produce TMA by the purified choline TMA lyase CutC/D, suggesting that the enzyme may only recognize the choline moiety, either in its free or conjugated forms. The enzymes phospholipase D, intestinal alkaline phosphatase, and glycerophosphodiester phosphodiesterase are secreted from pancreas or intestinal mucosal cells and may further contribute to the hydrolysis of GPC to produce choline [57]. Choline has been shown to promote atherosclerosis in a TMAO-dependent manner [14], and here we show that GPC can follow the same metaorganismal pathway.

Butyric acid is a major energy source for colonic epithelial cells [58] and can upregulate the expression of intestinal tight junction proteins—thus promoting gut barrier integrity and decreasing the production of pro-inflammatory cytokines induced by the endotoxin lipopolysaccharide (LPS) [59]. GPC supplementation shifts the gut microbial community, leading to a decreased abundance of butyric acid-producing bacteria and consequently butyric acid. Decreased butyric acid levels may further contribute to the progression of atherosclerosis in GPC-fed mice.

GPC supplementation decreases the abundance of several beneficial microbes such as *Akkermansia*, which has been implicated in the enhancement of gut barrier integrity, dietary interventions, and cancer immunotherapy [60,61,62,63]. The oral gavage of *Akkermansia* attenuates atherosclerotic lesion formation in western diet-induced *Apoe*^−/−^ mice and prebiotics such as berberine can increase the abundance of *Akkermansia* in gut to attenuate atherosclerosis [64,65]. Additionally, we reported that GPC decreases the abundance of *Lactobacillus* in the mouse gut. *Lactobacillus* is a widely investigated probiotic, which has been shown to strengthen gut barrier integrity, thus decreasing bacterial translocation and circulating endotoxin levels [66]. The endotoxin LPS can interact with TLR4 to trigger a cytokine cascade, leading to the development of atherosclerosis [67].

Increased levels of plasma CXCL13, also known as B-cell-attracting chemokine 1 (BCA1), were previously reported in patients with carotid atherosclerosis. CXCR5 is the receptor for CXCL13, and both the deficiency of CXCR5 and CXCL13 have been shown to attenuate atherosclerosis [68], thus underscoring a clear role for CXCL13 signaling in atherosclerosis. In this study, we report that GPC supplementation increases the expression of CXCL13. Similarly, tissue inhibitors of metalloproteinases 1 (TIMP-1) have been associated with atherosclerotic plaque formation and arterial calcification, thus contributing to the development and progression of atherosclerosis [69,70]. Interestingly, we found that GPC supplementation leads to a marked increase in TIMP-1 expression.

In a survey of the plasma metabolome, we were surprised to find that most of the metabolites that increase with GPC supplementation and are positively correlated with TMAO also positively associate with aortic lesion size. The observation that several metabolites strongly correlate with aortic lesion area but not TMAO suggests that other metabolic pathways mediating GPC-induced atherosclerosis may exist outside of the TMAO metaorganismal pathway.

In conclusion, the dietary nutrient GPC can promote atherosclerosis through the TMAO metaorganismal pathway as well as other molecular mechanisms. Further studies are needed to confirm these recent findings with larger clinical cohorts. Based on the current findings and the newly reported association between GPC consumption and increased 10-year incident stroke risk [13], caution should be applied when using GPC as a nutritional supplement.

## 4. Materials and Methods

### 4.1. Reagents

d_9_-trimethylamine N-oxide (d_9_-TMAO), d_9_-[N,N,N-trimethyl] choline (d_9_-choline), and d_9_-trimethylamine (d_9_-TMA) were purchased from Cambridge Isotope Laboratories (Andover, MA). d_6_-choline [(N,N-dimethyl-d_6_)-choline, iodide salt] and [N,N,N-trimethyl-d_9_] glycerylphosphorylcholine (d_9_-GPC) were chemically synthesized and purified in our lab, as previously reported [21,71]. [N,N,N-trimethyl-d_9_]-betaine (d_9_-betaine) was purchased from C/D/N ISOTOPES INC (Quebec, Canada). Rabbit polyclonal antibodies that recognize beta-GAPDH (#14C10), p44/42 MAPK (Erk1/2) (#4695S), phospho-p44/42 MAPK (Erk1/2) (Thr202/Tyr204) (#4370S), p38 MAPK (#8690S), phospho-p38 MAPK (#9211S), NFκB p65 (# 8242S), and phospho-NFκB p65 (Ser536) (# 3033S) were purchased from Cell Signaling Technology (Danvers, MA, USA). Horseradish peroxidase (HRP)–conjugated secondary antibodies that recognize rabbit IgG (#32460) was purchased from Thermo Fisher (Waltham, MA, USA). All the solvents for mass spectrometry were purchased from Fisher Scientific (Waltham, MA, USA), with HPLC grade and the other reagents purchased from Sigma, unless otherwise specified.

### 4.2. General Procedures

Plasma glucose and lipid profiles were measured on the Abbott ARCHITECT platform (Abbott Diagnostics, Abbott Park, IL, USA). Protein concentration was determined by Pierce™ BCA Protein Assay Kit (ThermoFisher Scientific, Waltham, MA, USA). Cecal bacterial DNA was isolated by QIAamp PowerFecal DNA kit (CAT# 12830, QIAGEN, Germantown, MD, USA). Chemokines and cytokines were assayed by ELISA kit (R&D Systems, CAT# MTM100 and MCX130 for TIMP1 and CXCL13/BLC/BCA-1, respectively).

### 4.3. Human Coronary Artery Endothelial Cells (HCAECs) Culture

HCAEC (ATCC^®^ PCS-100-020, Manassas, VA, USA) were seeded in 6-well plates with 1.5 × 10^5^ per well and cultured in Vascular Cell Basal Medium (ATCC^®^ PCS-100-030) supplemented with 20% FBS, Endothelial Cell Growth Kit-VEGF (ATCC^®^ PCS-100-041), until a confluency of approximately 60%. Cells were serum starved overnight in culture medium supplemented with 2% FBS, then different concentrations of GPC were added. Twenty minutes later, cells were harvested and lysed in RIPA buffer (ThermoFisher Scientific, Waltham, MA, USA) with protease inhibitors, PMSF, and phosphatase inhibitor, PhosSTOP™ (Sigma-Aldrich, St. Louis, MO, USA) was added.

### 4.4. SDS-PAGE/Western Blotting

A portion of 4–12% gradient gel was used to resolve proteins. Each well was loaded with 4 µg protein equivalent of cell lysate, and 1 µg protein marker (Bio-Rad, #1610374, Hercules, CA, USA) was loaded to a spare well. The gel was run in SDS-PAGE running buffer (Bio-Rad, #1610772) at a voltage of 100. After electrophoresis, the gels were blotted to PVDF membranes using Bio-Rad Western Blotting Transfer Cell with Tris/Glycine Buffer (Bio-Rad, #1610734). Immunoblot after incubation with the indicated primary antibody was probed with an HRP-conjugated secondary antibody and then incubated with SuperSignal™ West Dura Extended Duration Substrate (#34075, Thermo Scientific). A picture was taken under Amersham Imager 600 (GEHealthcare, Chicago, IL, USA), and band intensity was quantified by ImageJ software (National Institutes of Health).

### 4.5. Proteome Profiler^TM^ Array

Mouse Cytokine Array Panel A kit (CAT# ARY006, R&D Systems, Inc., Minneapolis, MN, USA) was used to detect the relative levels of cytokines and chemokines in mouse plasma, as directed by the manufacturer. Membranes after incubation with the Chemi Reagent Mix (included in the kit) were imaged by using Amersham Imager 600 system (GEHealthcare, Chicago, IL, USA), and a densitometric analysis of the intensities of the cytokine dots was performed by ImageJ software (National Institutes of Health).

### 4.6. Animals

C57BL/6J, Apolipoprotein E knockout mice (C57BL/6J.*Apoe*^−/−^) and LDL receptor knockout mice (C57BL/6J.*LDLr*^−/−^) were purchased from The Jackson Laboratory (Bar Harbor, ME, USA) and bred in the Biological Research Unit of Lerner Research Institute according to the approved IACUC protocol. Animals were maintained in ventilated microisolator cages under a strict 14 h light/10 h dark cycle, ambient temperature 20–26 °C, and humidity 30–70%, with water and food (TD. 2918, Envigo, Indianapolis, IN, USA), available ad libitum. GPC and choline were dissolved in the drinking water at a concentration of 1.0%.

### 4.7. LC-MS/MS Quantitation of Plasma and Tissue Metabolites

Plasma was mixed with 4 volumes of methanol containing stable isotope labeled compounds as internal standard. Following centrifugation at 20,000× *g*, 4 °C for 10 min, the supernatant was injected onto the LC-MS for metabolite monitoring and quantification. Murine intestinal segments were homogenized with 10 volumes of isotope labeled internal standard mix in water followed by centrifugation at 2500× *g*, 4 °C, for 10 min, and a half volume of the supernatant was filtered through a 3K cut-off membrane (Ref# UFC500396, Amicon Ultra, Millipore, Temecula, CA, USA) for short chain fatty acid assay, and the other half of the supernatant was used for TMA assay. The cecal content filtrate for short chain fatty acid quantitation was derivatized with 3-nitrophenylhydrazine, following the protocol as reported in [72]. The supernatant for TMA assay followed hexane/butanol extraction under alkaline pH, as previously reported for urine TMA assay [73]. The supernatant or filtrate for other metabolites was directly injected onto Shimadzu 8050 LC-MS (Kyoto, Japan) using Luna^®^ Silica column (150 × 2 mm, #00F-4274-B0, Phenomenx, Torrance, CA, USA) with LC gradient generated from two solvents: 0.1% propionic acid in water and 0.1% acetic acid in methanol. The metabolites were monitored by a multiple reaction monitoring mechanism with specific parent to daughter transition corresponding to each metabolite in positive or negative mode, and the MS parameters were optimized by individual metabolite. Serial dilutions of standards undergoing the same sample processing were used to prepare standard curves.

### 4.8. Untargeted Metabolomics of Mouse Plasma Samples

Samples were analyzed on a Triple TOF™ 5600 System (AB Sciex^®^, Framingham, MA, USA) in positive IDA mode operated by Analyst^®^ 1.7 software. The mass spectrometer was set to full spectra scan (*m*/*z* 50–1000) for 250 ms, followed by an MS/MS scan from the top 10 abundant ions in each cycle. The accumulation time for each IDA experiment was 50 ms, and the CE was set to 25 eV. Luna^®^ Silica column (150 × 2 mm, #00F-4274-B0, Phenomenx) was used to resolve metabolites with a gradient composed of A, 10 mM ammonium acetate in water; B, 0.1% acetic acid in 50% methanol and 50% acetonitrile, generated by a binary Shimadzu LC-20AD pumps. The first 2 min were set to 0% B, then linearly increased to 100% B within 25 min and held at 100% B for 5 min, then back to 0% B followed by a 3 min equilibration. The flow rate was 0.2 mL/min, and the eluate from the column in the first 2 min was diverted to waste. The total data acquisition window is 28 min. After precipitating proteins with 4 volumes of methanol, 3 µL plasma supernatant was injected onto the column via an autosampler (SIL-20ACXR, Shimadzu, Kyoto, Japan).

### 4.9. Metabolic Challenges in Mice

C57BL/6J *Apoe*^−/−^ mice were given an oral gavage containing stable-isotope-labelled d_9_-GPC using a 1.5-inch 20-gauge feeding needle. The gavage consisted of 150 µL of 150 mM. Blood (20 µL) was collected via the saphenous vein from mice at baseline and after gavage at different time points.

### 4.10. Intestinal Segment Incubation with d_9_-GPC and d_9_-Choline

Five female and three male C57BL/6J mice were humanely euthanized and the intestine was cut into different segments: duodenum, jejunum, ileum, cecum, and colon. Each intestinal segment was weighed in a balance with a resolution of 0.1 mg and was cut into different pieces around 0.5–1 cm in length, where one half was used for incubation with d_9_-choline and the other half was used for incubation with d_9_-GPC. 20 volumes (1 mg tissue is estimated to 1 µL in volume) of 50 µM d9-GPC, and 50 µM d9-choline in 10 mM Hepes buffer (pH 7.4) were added to incubate the intestinal segment at 37 °C. Sixteen hours later, the reaction was stopped by adding 1% volume of 6 N HCl. After centrifugation followed by filtration through the 3K cut-off membrane, the supernatant was collected to quantify d_9_-choline, d_9_-GPC, and d_9_-TMA with 10 µM choline chloride-1,1,2,2-d_4_ (d_4_-choline) and [^13^C_3_,^15^N]TMA added as internal standard.

### 4.11. Bacterial Culture

*Proteus mirabilis* (ATCC 29906™), *Escherichia fergusonii* (ATCC 35469™), *Lactobacillus acidophilus* (ATCC 4356), and *Escherichia coli* strain Top10 (Invitrogen) were grown in LB medium in an incubator shaking at 250 rpm, 37 °C overnight. The bacterial suspension was spun down at 6000× *g*, 4 °C for 12 min and resuspended in ¼ volume of cold PBS for incubation with d_9_-GPC and d_6_-choline at a final concentration of 100 µM. *P. mirabilis* lysate was prepared by Microfluidizer (M110Y, Microfluidics Corporation, Newton, MA, USA), with lysozyme added in advance for incubation with d_9_-GPC and d_9_-choline.

### 4.12. Aortic Root Lesion Area Quantification

Apolipoprotein E knockout mice (C57BL/6J.*Apoe*^−/−^) were weaned at 4 weeks of age and fed a standard chow diet (TD. 2918, Envigo, TD. 2918, Indianapolis, IN, USA), with 1.0% GPC added to the drinking water or regular drinking water. Mice were anesthetized with 300 mg/kg Ketamine + 30 mg/kg Xylazine prior to cardiac puncture for blood collection. The circulatory system was then perfused by intraventricular injection twice with 10 mL cold PBS prior to collection of the heart. Hearts were fixed and stored in 4% paraformaldehyde prior to frozen OCT sectioning, and the OCT section (6 µm thickness) was stained with Oil-Red-O and haematoxylin. Aortic root lesion area was quantified as the mean value of 6 sections [74].

### 4.13. Real Time (RT)-PCR

RT-PCR of cecal bacteria encoding the choline TMA lyase *cutC* was performed using Brilliant II SYBR^®^ Green QRT-PCR kit (#600834, Strategene, Bellingham, WA, USA). The forward and reverse primers, *cut*C and 16S rRNA, were synthesized by Integrated DNA Technologies, Inc. (IDT, Coralville, IA, USA) based on sequences reported in references [43]. iTaq™ Universal SYBR^®^ Green Supermix (Bio-Rad, # 1725122) was used to conduct the PCR reaction in a 96-well plate, with 50 ng DNA added as template in each well. RT-PCR was done in StepOnePlus™ Real-Time PCR System (Applied Biosystems) with initial denaturation at 95 °C for 3 min, followed by 40 cycles of denaturation at 95 °C for 15 s and annealing and elongation at 60 °C for 1 min. The PCR products were confirmed by melting curves with denaturation ramp between 60 °C and 95 °C at 0.3 °C per minute, and the specificity of qPCR reaction was further confirmed by agarose gel electrophoresis. The 2^−ΔΔCt^ method was used for the quantification of *cutC* gene with 16S rRNA gene as reference.

### 4.14. Determination of TMA Lyase Activity

Cecal and fecal bacterial TMA lyase activity was determined by incubation of deuterium labeled chemicals with a structural formula containing TMA under anaerobic condition, as previously described [21]. Incubation was conducted under anaerobic condition, either by filling with argon (Praxair, Morrisville, PA, USA) or in an anaerobic chamber (Coy Lab Products, Grass Lake, MI, USA) filled with N_2_ and H_2_.

### 4.15. Microbiota Profiling by 16S rRNA Gene Sequencing

Bacterial DNA was extracted from mouse cecum using the PowerSoil DNA Isolation Kit (#12888, MO BIO Laboratories, Carlsbad, CA, USA). Amplification and sequencing of the V4 hypervariable region of the 16S rRNA gene was performed using the region-specific bacterial primers 515F and 806R. The PCR conditions consisted of an initial denaturation step of 94 °C for 3 min; 35 cycles of 94 °C for 45 s, 50 °C for 30 s, and 72 °C for 90 s, followed by 72 °C for 5 min. Replicate amplicons were quantified with Quant-iT™ PicoGreen^®^ dsDNA Assay Kit (Life Technologies Corporation, Carlsbad, CA) according to the manufacturer’s instructions, and pooled and purified using the UltraClean^®^ PCR Clean-up Kit (MO BIO Laboratories, Inc., Carlsbad, CA, USA) according to manufacturer’s instructions. High-throughput sequencing analysis of bacterial rRNA genes was performed on the purified, pooled sample using the Illumina MisSeq platform at the UCLA Genotyping and Sequencing Core, University of California, Los Angeles, as previously described [75]. De-multiplexing 16S rRNA gene sequences, quality control, and operational taxonomic unit (OTU) binning were performed using open source pipeline Quantitative Insights into Microbial Ecology (QIIME) version 1.7.0 [76], using established guidelines [77]. Quality-filtered reads were de-multiplexed, and the total number of sequencing reads were 459,625 (an average of 21,887 reads per sample) with an average length of 153 base pair reads. Sequences were binned into Operational Taxonomic Units (OTUs) based on 97% identity using UCLUST [78] against the Greengenes reference database (version gg_13_8). Each sample’s sequences were rarefied to 16,717 reads per sample to reduce the effect of sequencing depth.

### 4.16. Human Fecal Samples

Human fecal samples were collected from subjects participating in the Healthy Life in an Urban Setting (HELIUS) study, as previously reported [79]. This study has been approved by the AMC institutional review board (Approval ID: MREC 10/100# 17.10.1729), and all participants provided written informed consent.

### 4.17. Statistical Analyses

Student’s *t* test, Wilcoxon rank sum test, or one-way ANOVA was used to determine statistical significance where appropriate. Spearman’s or Pearson’s correlation was used to calculate the association between two variables. Plots of normalized concentration were drawn by the R program (version 4.0.5) with the ggplot2 package installed. Microbial composition at each taxonomic level was defined using the summarize taxa function in QIIME. Alpha diversity and beta-diversity (weighted UniFrac metrics) were calculated using MicrobiomeAnalyst [80,81]. LEfSe (linear discriminant analysis (LDA) coupled with effect size measurements) was calculated by a web-based genome analysis tool, Galaxy, which was provided by the Huttenhower Lab [82] under the following conditions: (1) the alpha value for the factorial Kruskal–Wallis test among classes is <0.05 and (2) the threshold on the logarithmic LDA score for discriminative features is >2.0. The untargeted metabolomics files generated by Triple-TOF 5600 mass spectrometer, wiff and wiff.scan files, were loaded into XCMS Online [83] to extract ion specific features labeled with m/z and retention time followed by comparison between GPC-fed mice and control chow-fed mice. The data from XCMS online were further analyzed by MetaboAnalyst 5.0, with the relative concentration in each sample normalized by sum and using data autoscaling [84]. For all statistical tests, *p* < 0.05 was considered significant.

## Figures and Tables

**Table 1:** Plasma metabolites and ALT, AST in C57BL/6J *Apoe*^−/−^ mice after being fed GPC supplemented chow diet vs. control diet for 16 weeks.

Parameter	Male		Female	
	Control (n = 21)	GPC (n = 11)	Control (n = 13)	GPC (n = 12)
TMAO (μM)	2.0 ± 0.6	35.4 ± 14.3 ***	13.1 ± 6.1	186.4 ± 85.7 ***
GPC (μM)	6.8 ± 3.3	7.3 ± 1.9	7.5 ± 2.1	7.9 ± 4.2
choline (μM)	26.4 ± 9.1	32.7 ± 10.7	14.5 ± 2.1	17.8 ± 3.9
cholesterol (mg/dL)	340 ± 93	368 ± 75	334 ± 46	324 ± 59
triglyceride (mg/dL)	105 ± 50	98 ± 35	100 ± 27	106 ± 31
creatinine (μM)	7.8 ± 1.1	8.1 ± 2.4	8.8 ± 1.4	8.8 ± 1.8
glucose (mg/dL)	277 ± 64	249 ± 73	263 ± 67	231 ± 59
ALT (u/L)	56.6 ± 26.9	33.1 ± 9.3	38.2 ± 21.0	35.7 ± 35.6
AST (u/L)	137 ± 56	113 ± 40	138 ± 150	173 ± 268

Diet was started after a weaning age of four weeks. ALT, alanine aminotransferase; AST, aspartate aminotransferase. *** *p* < 0.001 by comparison with control chow diet using Student’s *t* test.

## Data Availability

All data can be provided upon written request.

## References

[1] Amenta F., Tayebati S.K., Vitali D., Di Tullio M.A. (2006). Association with the cholinergic precursor choline alphoscerate and the cholinesterase inhibitor rivastigmine: An approach for enhancing cholinergic neurotransmission. Mech. Ageing Dev..

[2] Gatti G., Barzaghi N., Acuto G., Abbiati G., Fossati T., Perucca E. (1992). A comparative study of free plasma choline levels following intramuscular administration of L-alpha-glycerylphosphorylcholine and citicoline in normal volunteers. Int. J. Clin. Pharmacol. Ther. Toxicol..

[3] (2008). USDA Database for the Choline Content of Common Foods. https://www.ars.usda.gov/ARSUserFiles/80400525/Data/Choline/Choln02.pdf.

[4] Bang H.J., Kim I.H., Kim B.H. (2016). Phospholipase A1-catalyzed hydrolysis of soy phosphatidylcholine to prepare l-alpha-glycerylphosphorylcholine in organic-aqueous media. Food Chem..

[5] Song Y., Roh S., Hwang J., Chung M.Y., Kim I.H., Kim B.H. (2020). Immobilized Phospholipase A1-Catalyzed Preparation of l-alpha-Glycerylphosphorylcholine from Phosphatidylcholine. J. Agric. Food Chem..

[6] Van den Bosch H., Aarsman A.J., van Deenen L.L. (1974). Isolation and properties of a phospholipase A1 activity from beef pancreas. Biochim. Biophys. Acta.

[7] Zhao T., No da S., Kim B.H., Garcia H.S., Kim Y., Kim I.H. (2014). Immobilized phospholipase A1-catalyzed modification of phosphatidylcholine with n-3 polyunsaturated fatty acid. Food Chem..

[8] Van der Veen J.N., Kennelly J.P., Wan S., Vance J.E., Vance D.E., Jacobs R.L. (2017). The critical role of phosphatidylcholine and phosphatidylethanolamine metabolism in health and disease. Biochim. Biophys. Acta Biomembr..

[9] Lee S.H., Choi B.Y., Kim J.H., Kho A.R., Sohn M., Song H.K., Choi H.C., Suh S.W. (2017). Late treatment with choline alfoscerate (l-alpha glycerylphosphorylcholine, alpha-GPC) increases hippocampal neurogenesis and provides protection against seizure-induced neuronal death and cognitive impairment. Brain Res..

[10] Sigala S., Imperato A., Rizzonelli P., Casolini P., Missale C., Spano P. (1992). L-alpha-glycerylphosphorylcholine antagonizes scopolamine-induced amnesia and enhances hippocampal cholinergic transmission in the rat. Eur. J. Pharmacol..

[11] Marcus L., Soileau J., Judge L.W., Bellar D. (2017). Evaluation of the effects of two doses of alpha glycerylphosphorylcholine on physical and psychomotor performance. J. Int. Soc. Sports Nutr..

[12] Bellar D., LeBlanc N.R., Campbell B. (2015). The effect of 6 days of alpha glycerylphosphorylcholine on isometric strength. J. Int. Soc. Sports Nutr..

[13] Lee G., Choi S., Chang J., Choi D., Son J.S., Kim K., Kim S.M., Jeong S., Park S.M. (2021). Association of L-alpha Glycerylphosphorylcholine With Subsequent Stroke Risk After 10 Years. JAMA Netw. Open.

[14] Wang Z., Klipfell E., Bennett B.J., Koeth R., Levison B.S., Dugar B., Feldstein A.E., Britt E.B., Fu X., Chung Y.M. (2011). Gut flora metabolism of phosphatidylcholine promotes cardiovascular disease. Nature.

[15] Koeth R.A., Wang Z., Levison B.S., Buffa J.A., Org E., Sheehy B.T., Britt E.B., Fu X., Wu Y., Li L. (2013). Intestinal microbiota metabolism of L-carnitine, a nutrient in red meat, promotes atherosclerosis. Nat. Med..

[16] Koeth R.A., Levison B.S., Culley M.K., Buffa J.A., Wang Z., Gregory J.C., Org E., Wu Y., Li L., Smith J.D. (2014). gamma-Butyrobetaine is a proatherogenic intermediate in gut microbial metabolism of L-carnitine to TMAO. Cell Metab..

[17] Zhao Y., Wang Z. (2020). Impact of trimethylamine N-oxide (TMAO) metaorganismal pathway on cardiovascular disease. J. Lab. Precis. Med..

[18] Seldin M.M., Meng Y., Qi H., Zhu W., Wang Z., Hazen S.L., Lusis A.J., Shih D.M. (2016). Trimethylamine N-Oxide Promotes Vascular Inflammation Through Signaling of Mitogen-Activated Protein Kinase and Nuclear Factor-kappaB. J. Am. Heart Assoc..

[19] Haghikia A., Li X.S., Liman T.G., Bledau N., Schmidt D., Zimmermann F., Krankel N., Widera C., Sonnenschein K., Haghikia A. (2018). Gut Microbiota-Dependent Trimethylamine N-Oxide Predicts Risk of Cardiovascular Events in Patients with Stroke and Is Related to Proinflammatory Monocytes. Arterioscler. Thromb. Vasc. Biol..

[20] Donaldson G.P., Lee S.M., Mazmanian S.K. (2016). Gut biogeography of the bacterial microbiota. Nat. Rev. Microbiol..

[21] Wang Z., Roberts A.B., Buffa J.A., Levison B.S., Zhu W., Org E., Gu X., Huang Y., Zamanian-Daryoush M., Culley M.K. (2015). Non-lethal Inhibition of Gut Microbial Trimethylamine Production for the Treatment of Atherosclerosis. Cell.

[22] Craciun S., Balskus E.P. (2012). Microbial conversion of choline to trimethylamine requires a glycyl radical enzyme. Proc. Natl. Acad. Sci. USA.

[23] Roberts A.B., Gu X., Buffa J.A., Hurd A.G., Wang Z., Zhu W., Gupta N., Skye S.M., Cody D.B., Levison B.S. (2018). Development of a gut microbe-targeted nonlethal therapeutic to inhibit thrombosis potential. Nat. Med..

[24] Oppi S., Luscher T.F., Stein S. (2019). Mouse Models for Atherosclerosis Research-Which Is My Line?. Front. Cardiovasc. Med..

[25] Getz G.S., Reardon C.A. (2012). Animal models of atherosclerosis. Arterioscler Thromb. Vasc. Biol..

[26] Lo Sasso G., Schlage W.K., Boue S., Veljkovic E., Peitsch M.C., Hoeng J. (2016). The Apoe(−/−) mouse model: A suitable model to study cardiovascular and respiratory diseases in the context of cigarette smoke exposure and harm reduction. J. Transl. Med..

[27] Bennett B.J., de Aguiar Vallim T.Q., Wang Z., Shih D.M., Meng Y., Gregory J., Allayee H., Lee R., Graham M., Crooke R. (2013). Trimethylamine-N-oxide, a metabolite associated with atherosclerosis, exhibits complex genetic and dietary regulation. Cell Metab..

[28] Zhang X., Li Y., Yang P., Liu X., Lu L., Chen Y., Zhong X., Li Z., Liu H., Ou C. (2020). Trimethylamine-N-Oxide Promotes Vascular Calcification Through Activation of NLRP3 (Nucleotide-Binding Domain, Leucine-Rich-Containing Family, Pyrin Domain-Containing-3) Inflammasome and NF-kappaB (Nuclear Factor kappaB) Signals. Arterioscler. Thromb. Vasc. Biol..

[29] Li X., Geng J., Zhao J., Ni Q., Zhao C., Zheng Y., Chen X., Wang L. (2019). Trimethylamine N-Oxide Exacerbates Cardiac Fibrosis via Activating the NLRP3 Inflammasome. Front. Physiol..

[30] El-Deeb O.S., Atef M.M., Hafez Y.M. (2019). The interplay between microbiota-dependent metabolite trimethylamine N-oxide, Transforming growth factor beta/SMAD signaling and inflammasome activation in chronic kidney disease patients: A new mechanistic perspective. J. Cell Biochem..

[31] Boini K.M., Hussain T., Li P.L., Koka S. (2017). Trimethylamine-N-Oxide Instigates NLRP3 Inflammasome Activation and Endothelial Dysfunction. Cell Physiol. Biochem..

[32] Chen M.L., Zhu X.H., Ran L., Lang H.D., Yi L., Mi M.T. (2017). Trimethylamine-N-Oxide Induces Vascular Inflammation by Activating the NLRP3 Inflammasome Through the SIRT3-SOD2-mtROS Signaling Pathway. J. Am. Heart Assoc..

[33] Yue C., Yang X., Li J., Chen X., Zhao X., Chen Y., Wen Y. (2017). Trimethylamine N-oxide prime NLRP3 inflammasome via inhibiting ATG16L1-induced autophagy in colonic epithelial cells. Biochem. Biophys. Res. Commun..

[34] Sun X., Jiao X., Ma Y., Liu Y., Zhang L., He Y., Chen Y. (2016). Trimethylamine N-oxide induces inflammation and endothelial dysfunction in human umbilical vein endothelial cells via activating ROS-TXNIP-NLRP3 inflammasome. Biochem. Biophys. Res. Commun..

[35] Bai Y., Zhao J.B., Tao S.Y., Zhou X.J., Pi Y., Gerrits W.J., Johnston L.J., Zhang S.Y., Yang H.J., Liu L. (2020). Effect of dietary fiber fermentation on short-chain fatty acid production and microbial composition in vitro. J. Sci. Food Agric..

[36] Mortensen P.B., Holtug K., Rasmussen H.S. (1988). Short-chain fatty acid production from mono- and disaccharides in a fecal incubation system: Implications for colonic fermentation of dietary fiber in humans. J. Nutr..

[37] Yao Y., Cai X., Fei W., Ye Y., Zhao M., Zheng C. (2020). The role of short-chain fatty acids in immunity, inflammation and metabolism. Crit Rev. Food Sci Nutr..

[38] Robles-Vera I., Toral M., de la Visitacion N., Aguilera-Sanchez N., Redondo J.M., Duarte J. (2020). Protective Effects of Short-Chain Fatty Acids on Endothelial Dysfunction Induced by Angiotensin II. Front. Physiol..

[39] Esper R.J., Nordaby R.A., Vilarino J.O., Paragano A., Cacharron J.L., Machado R.A. (2006). Endothelial dysfunction: A comprehensive appraisal. Cardiovasc. Diabetol..

[40] Duncan S.H., Hold G.L., Barcenilla A., Stewart C.S., Flint H.J. (2002). Roseburia intestinalis sp. nov., a novel saccharolytic, butyrate-producing bacterium from human faeces. Int. J. Syst. Evol. Microbiol..

[41] Machiels K., Joossens M., Sabino J., De Preter V., Arijs I., Eeckhaut V., Ballet V., Claes K., Van Immerseel F., Verbeke K. (2014). A decrease of the butyrate-producing species Roseburia hominis and Faecalibacterium prausnitzii defines dysbiosis in patients with ulcerative colitis. Gut.

[42] Jiang S., Xie S., Lv D., Zhang Y., Deng J., Zeng L., Chen Y. (2016). A reduction in the butyrate producing species Roseburia spp. and Faecalibacterium prausnitzii is associated with chronic kidney disease progression. Antonie Van Leeuwenhoek.

[43] Martinez-del Campo A., Bodea S., Hamer H.A., Marks J.A., Haiser H.J., Turnbaugh P.J., Balskus E.P. (2015). Characterization and detection of a widely distributed gene cluster that predicts anaerobic choline utilization by human gut bacteria. mBio.

[44] Shih D.M., Wang Z., Lee R., Meng Y., Che N., Charugundla S., Qi H., Wu J., Pan C., Brown J.M. (2015). Flavin containing monooxygenase 3 exerts broad effects on glucose and lipid metabolism and atherosclerosis. J. Lipid Res..

[45] Stubbs J.R., House J.A., Ocque A.J., Zhang S., Johnson C., Kimber C., Schmidt K., Gupta A., Wetmore J.B., Nolin T.D. (2016). Serum Trimethylamine-N-Oxide is Elevated in CKD and Correlates with Coronary Atherosclerosis Burden. J. Am. Soc. Nephrol..

[46] Randrianarisoa E., Lehn-Stefan A., Wang X., Hoene M., Peter A., Heinzmann S.S., Zhao X., Konigsrainer I., Konigsrainer A., Balletshofer B. (2016). Relationship of Serum Trimethylamine N-Oxide (TMAO) Levels with early Atherosclerosis in Humans. Sci. Rep..

[47] Mente A., Chalcraft K., Ak H., Davis A.D., Lonn E., Miller R., Potter M.A., Yusuf S., Anand S.S., McQueen M.J. (2015). The Relationship Between Trimethylamine-N-Oxide and Prevalent Cardiovascular Disease in a Multiethnic Population Living in Canada. Can. J. Cardiol..

[48] Kim R.B., Morse B.L., Djurdjev O., Tang M., Muirhead N., Barrett B., Holmes D.T., Madore F., Clase C.M., Rigatto C. (2016). Advanced chronic kidney disease populations have elevated trimethylamine N-oxide levels associated with increased cardiovascular events. Kidney Int.

[49] Roncal C., Martinez-Aguilar E., Orbe J., Ravassa S., Fernandez-Montero A., Saenz-Pipaon G., Ugarte A., Estella-Hermoso de Mendoza A., Rodriguez J.A., Fernandez-Alonso S. (2019). Trimethylamine-N-Oxide (TMAO) Predicts Cardiovascular Mortality in Peripheral Artery Disease. Sci. Rep..

[50] Zheng L., Zheng J., Xie Y., Li Z., Guo X., Sun G., Sun Z., Xing F., Sun Y. (2019). Serum gut microbe-dependent trimethylamine N-oxide improves the prediction of future cardiovascular disease in a community-based general population. Atherosclerosis.

[51] Tang W.W., Wang Z., Levison B.S., Koeth R.A., Britt E.B., Fu X., Wu Y., Hazen S.L. (2013). Intestinal microbial metabolism of phosphatidylcholine and cardiovascular risk. N. Engl. J. Med..

[52] Troseid M., Ueland T., Hov J.R., Svardal A., Gregersen I., Dahl C.P., Aakhus S., Gude E., Bjorndal B., Halvorsen B. (2015). Microbiota-dependent metabolite trimethylamine-N-oxide is associated with disease severity and survival of patients with chronic heart failure. J. Intern. Med..

[53] Suzuki T., Yazaki Y., Voors A.A., Jones D.J.L., Chan D.C.S., Anker S.D., Cleland J.G., Dickstein K., Filippatos G., Hillege H.L. (2019). Association with outcomes and response to treatment of trimethylamine N-oxide in heart failure: Results from BIOSTAT-CHF. Eur. J. Heart Fail..

[54] Dong Z., Liang Z., Wang X., Liu W., Zhao L., Wang S., Hai X., Yu K. (2020). The correlation between plasma trimethylamine N-oxide level and heart failure classification in northern Chinese patients. Ann. Palliat. Med..

[55] Li W., Huang A., Zhu H., Liu X., Huang X., Huang Y., Cai X., Lu J., Huang Y. (2020). Gut microbiota-derived trimethylamine N-oxide is associated with poor prognosis in patients with heart failure. Med. J. Aust..

[56] Zhou X., Jin M., Liu L., Yu Z., Lu X., Zhang H. (2020). Trimethylamine N-oxide and cardiovascular outcomes in patients with chronic heart failure after myocardial infarction. ESC Heart Fail..

[57] Nilsson A., Duan R.D. (2019). Pancreatic and mucosal enzymes in choline phospholipid digestion. Am. J. Physiol. Gastrointest. Liver Physiol..

[58] Rowe W.A., Bayless T.M. (1992). Colonic short-chain fatty acids: Fuel from the lumen?. Gastroenterology.

[59] Bai H.B., Yang P., Zhang H.B., Liu Y.L., Fang S.X., Xu X.Y. (2021). Short-chain fatty acid butyrate acid attenuates atherosclerotic plaque formation in apolipoprotein E-knockout mice and the underlying mechanism. Sheng Li Xue Bao.

[60] Naito Y., Uchiyama K., Takagi T. (2018). A next-generation beneficial microbe: Akkermansia muciniphila. J. Clin. Biochem. Nutr..

[61] Plovier H., Everard A., Druart C., Depommier C., Van Hul M., Geurts L., Chilloux J., Ottman N., Duparc T., Lichtenstein L. (2017). A purified membrane protein from Akkermansia muciniphila or the pasteurized bacterium improves metabolism in obese and diabetic mice. Nat. Med..

[62] Reunanen J., Kainulainen V., Huuskonen L., Ottman N., Belzer C., Huhtinen H., de Vos W.M., Satokari R. (2015). Akkermansia muciniphila Adheres to Enterocytes and Strengthens the Integrity of the Epithelial Cell Layer. Appl. Environ. Microbiol..

[63] Roopchand D.E., Carmody R.N., Kuhn P., Moskal K., Rojas-Silva P., Turnbaugh P.J., Raskin I. (2015). Dietary Polyphenols Promote Growth of the Gut Bacterium Akkermansia muciniphila and Attenuate High-Fat Diet-Induced Metabolic Syndrome. Diabetes.

[64] Zhu L., Zhang D., Zhu H., Zhu J., Weng S., Dong L., Liu T., Hu Y., Shen X. (2018). Berberine treatment increases Akkermansia in the gut and improves high-fat diet-induced atherosclerosis in Apoe(−/−) mice. Atherosclerosis.

[65] Li J., Lin S., Vanhoutte P.M., Woo C.W., Xu A. (2016). Akkermansia Muciniphila Protects Against Atherosclerosis by Preventing Metabolic Endotoxemia-Induced Inflammation in Apoe−/− Mice. Circulation.

[66] Nissen L., Chingwaru W., Sgorbati B., Biavati B., Cencic A. (2009). Gut health promoting activity of new putative probiotic/protective Lactobacillus spp. strains: A functional study in the small intestinal cell model. Int. J. Food Microbiol..

[67] Konev Iu V., Lazebnik L.B. (2011). Endotoxin (LPS) in the pathogenesis of atherosclerosis. Eksp. Klin. Gastroenterol..

[68] Van der Vorst E.P.C., Daissormont I., Aslani M., Seijkens T., Wijnands E., Lutgens E., Duchene J., Santovito D., Doring Y., Halvorsen B. (2020). Interruption of the CXCL13/CXCR5 Chemokine Axis Enhances Plasma IgM Levels and Attenuates Atherosclerosis Development. Thromb. Haemost..

[69] Zureik M., Beaudeux J.L., Courbon D., Benetos A., Ducimetiere P. (2005). Serum tissue inhibitors of metalloproteinases 1 (TIMP-1) and carotid atherosclerosis and aortic arterial stiffness. J. Hypertens.

[70] Orbe J., Fernandez L., Rodriguez J.A., Rabago G., Belzunce M., Monasterio A., Roncal C., Paramo J.A. (2003). Different expression of MMPs/TIMP-1 in human atherosclerotic lesions. Relation to plaque features and vascular bed. Atherosclerosis.

[71] Wang Z., Bergeron N., Levison B.S., Li X.S., Chiu S., Jia X., Koeth R.A., Li L., Wu Y., Tang W.H.W. (2019). Impact of chronic dietary red meat, white meat, or non-meat protein on trimethylamine N-oxide metabolism and renal excretion in healthy men and women. Eur. Heart J..

[72] Han J., Lin K., Sequeira C., Borchers C.H. (2015). An isotope-labeled chemical derivatization method for the quantitation of short-chain fatty acids in human feces by liquid chromatography-tandem mass spectrometry. Anal. Chim. Acta..

[73] Jia X., Osborn L.J., Wang Z. (2020). Simultaneous Measurement of Urinary Trimethylamine (TMA) and Trimethylamine N-Oxide (TMAO) by Liquid Chromatography-Mass Spectrometry. Molecules.

[74] Baglione J., Smith J.D. (2006). Quantitative assay for mouse atherosclerosis in the aortic root. Methods Mol. Med..

[75] Org E., Parks B.W., Joo J.W., Emert B., Schwartzman W., Kang E.Y., Mehrabian M., Pan C., Knight R., Gunsalus R. (2015). Genetic and environmental control of host-gut microbiota interactions. Genome Res..

[76] Caporaso J.G., Kuczynski J., Stombaugh J., Bittinger K., Bushman F.D., Costello E.K., Fierer N., Pena A.G., Goodrich J.K., Gordon J.I. (2010). QIIME allows analysis of high-throughput community sequencing data. Nat. Methods.

[77] Bokulich N.A., Subramanian S., Faith J.J., Gevers D., Gordon J.I., Knight R., Mills D.A., Caporaso J.G. (2013). Quality-filtering vastly improves diversity estimates from Illumina amplicon sequencing. Nat. Methods.

[78] Edgar R.C. (2010). Search and clustering orders of magnitude faster than BLAST. Bioinformatics.

[79] Deschasaux M., Bouter K.E., Prodan A., Levin E., Groen A.K., Herrema H., Tremaroli V., Bakker G.J., Attaye I., Pinto-Sietsma S.J. (2018). Depicting the composition of gut microbiota in a population with varied ethnic origins but shared geography. Nat. Med..

[80] Chong J., Liu P., Zhou G., Xia J. (2020). Using MicrobiomeAnalyst for comprehensive statistical, functional, and meta-analysis of microbiome data. Nat. Protoc..

[81] Dhariwal A., Chong J., Habib S., King I.L., Agellon L.B., Xia J. (2017). MicrobiomeAnalyst: A web-based tool for comprehensive statistical, visual and meta-analysis of microbiome data. Nucleic Acids Res..

[82] Segata N., Izard J., Waldron L., Gevers D., Miropolsky L., Garrett W.S., Huttenhower C. (2011). Metagenomic biomarker discovery and explanation. Genome Biol..

[83] Tautenhahn R., Patti G.J., Rinehart D., Siuzdak G. (2012). XCMS Online: A web-based platform to process untargeted metabolomic data. Anal. Chem..

[84] Pang Z., Chong J., Zhou G., de Lima Morais D.A., Chang L., Barrette M., Gauthier C., Jacques P.E., Li S., Xia J. (2021). MetaboAnalyst 5.0: Narrowing the gap between raw spectra and functional insights. Nucleic Acids Res..

